# A New Light-Sensor System Affecting Cancer Cell Fate

**DOI:** 10.34133/bmr.0157

**Published:** 2025-03-05

**Authors:** Silvia Buonvino, Ilaria Arciero, Stefano Moretti, Egidio Iorio, Sonia Melino

**Affiliations:** ^1^ Departmental Faculty of Medicine, UniCamillus - Saint Camillus International University of Health and Medical Sciences, 00131 Rome, Italy.; ^2^ Department of Chemical Science and Technologies, University of Rome Tor Vergata, 00133 Rome, Italy.; ^3^ CORE Facilities, ISS - Istituto Superiore di Sanità, Rome, Italy.; ^4^ Department of Experimental Medicine, University of Rome Tor Vergata, 00133 Rome Italy.; ^5^ NAST Center, University of Rome Tor Vergata, Rome, Italy.

## Abstract

A new physiological photopolymerizing system with relevant effects on proteins and able to affect cancer cell fate was discovered here. The riboflavin–phosphocholine–light (RPL) system induces lysozyme (LYZ) photopolymerization in vitro, affecting the cell viability of cancer cells, in both 2-dimensional and 3-dimensional cell cultures. The RPL treatment of nontumoral, mesenchymal stem cells, or cancer cells shows a distinct behavior, depending on the ectopic presence of LYZ. Morphological changes and cellular aggregation of the cancer cells were induced by the treatment. The presence of both phosphocholine and high levels of LYZ expression at the breast cancer cell–cell interface seems to create a vulnerability for this new photodynamic system under visible light exposure. Further, we generated 2 new riboflavin–phosphocholine hydrogels (RPHy and RPHy-LYZ) by light-emitting diode exposure. A transdifferentiation into osteoblast-like cells of a triple-negative breast cancer cell line, embedded into RPHy, was detected, while cell death was observed using RPHy-LYZ*.* Our results reveal new properties of phosphocholine and LYZ with potential translational implications linked to the study of the transdifferentiation process of breast cancer cells and to therapeutical applications. The results highlight new aspects of the molecular mechanism by which riboflavin acts on cancer cells, paving the way for the use of the physiological expression levels of both phosphocholine and LYZ in selective therapies using the RPL system.

## Introduction

Riboflavin (R) is a molecule unique in its properties and exhibits relevant biological activity [1]. Flavins, including R, flavin mononucleotide (FMN), and flavin adenine dinucleotide (FAD), are nontoxic and water-soluble molecules, and these characteristics make them optimal for several applications, including medical treatments. R and, more in general, flavins are photosensitizers able to generate singlet oxygen and other reactive oxygen species in the presence of a light source and for this reason find applications in various fields of photodynamics (PD), including medicine, photocatalysis, organic synthesis, environmental protection, cosmetology, and the development of skin care products. Photodynamic therapy (PDT) is a medical treatment that involves the use of a light-sensitive drug, oxygen and a light source to selectively destroy abnormal cells. The process typically involves the administration of a photosensitizing agent, which is a light-sensitive drug, followed by exposure to a specific wavelength. Many types of light sources have been proposed, including halogens, xenon, fluorescent, light-emitting diodes (LEDs), and laser light sources. The interaction between the photosensitizer, light, and oxygen leads to the production of reactive oxygen species, causing localized cell damage and destruction of targeted abnormal cells, such as cancer cells by programmed cell death (apoptosis) [2]. PDT is used in the treatment of various medical conditions, such as certain types of cancer, skin disorders, and other abnormal cell growths. R has been suggested as photosensitizer in the treatment of hematogenous metastasis [3]. However, the extensive use of ultraviolet (UV) A light (315/320 to 400 nm) in this context may pose risks such as photoaging, skin pigmentation, and even skin cancer. This supports the idea of using blue light for therapeutic purposes, considering the photophysical properties of flavins, including blue light absorption, efficient intersystem crossing, and the long lifetime of the triplet state. These properties make flavins, including R and derivatives, potentially useful in the treatment of skin diseases, including skin cancer [4]. Furthermore, R has been proposed as an adjuvant in the treatment of skin cancer in combination with cisplatin. Additionally, it has been considered as a photosensitizer in the treatment of cervical cancer cells (HeLa) [5]. PDT, as noninvasive method, has been used in the treatment of localized cutaneous melanoma, particularly in its early stages when the tumor thickness is less than 1 mm. The combination of FMN with blue light is mentioned as having a destructive effect on various human melanoma (Mel MTP, Mel IL, Mel Z, and A375) and squamous carcinoma (SCC-13) cells [6,7], suggesting that PDT with FMN and blue light may hold promise as a therapeutic procedure for these types of skin tumors.

Although the final effect of PDT using R as photosensitizer on the tumors is known, the molecular mechanism of the selective action on cancer cells is not well understood.

We have investigated this aspect and analyzed the possibility of the combined effect of PD treatment with phosphocholine (P), which is a natural ammine that could act as a co-initiator in a photoinduced reaction by triplet-excited R molecules. The physiological production of P in humans has been associated with the onset of tumors, and it has been considered a diagnostic marker for cancers such as ovarian and breast cancer [8,9]. The activity and the expression levels of choline kinase, which is an enzyme involved in P synthesis, were found to be significantly increased in human breast carcinoma cells [10], and this up-regulation is crucial for the proliferation of human mammary epithelial cells (HMECs) and breast tumor growth [11]. The most noticeable difference between normal HMECs and cancer cells was a 5- to 17-fold increase in P levels in tumor cell lines compared to those in normal mammary epithelial cells. Furthermore, additional results clearly demonstrated significant variations in P levels, which correlated with the maximum choline transport rate. The accumulation of high levels of P is thus determined by the high activity of choline kinase, suggesting that the presence of a high concentration of P in proliferating breast carcinoma cells can serve as a biomarker for breast malignancy transformation [9]. Therefore, we have investigated if a PD mechanism linked to the physiological P concentration could lead to a selective effect on the tumor cells.

The effects of the riboflavin–phosphocholine–light (RPL) system on the cell viability of normal, stem, and cancer cells were investigated here, observing a different behavior depending on the cell type. In particular, treatment with riboflavin–phosphocholine (RP) did not affect cell viability in standard conditions of cell growth, but in the presence of visible light (L, LED exposure), a significant decrease in cell viability only in the case of fibroblasts and breast cancer cells of the MDA-MB 231 cell line was observed here. This effect was also evaluated in 3-dimensional (3D) cell culture systems using tumoroids. Confocal fluorescence microscopy was used here to analyze the effects of the treatment using thioflavin S (ThS) and immunostaining. Recent studies have highlighted a correlation between high levels of lysozyme (LYZ) expression and cancer progression in different types of tumors, so LYZ has been indicated as a potential tumor biomarker [12–14]. In agreement, we investigated whether the selective effect of the RPL system can be related to the different levels of LYZ expression on the cell surface in different cell types.

Moreover, R, for its unique molecular properties, creates extensive opportunities for producing functional materials through polymerization processes. Recently, the use of hydrogels in cancer treatment is emerging as a new approach to addressing the limitations of traditional cancer therapies [15,16]. Hydrogels encapsulating antitumor drugs can provide a localized drug delivery specifically to the target site and, when combined with photosensitizers or hyperthermia-inducing agents, can be used for PD or photothermal therapy, respectively [15,16]. In this study, on the basis of the effects observed using the RPL system, a new polyethylene glycol diacrylate (PEGDA)-based hydrogel was obtained. This new photopolymerization process, using LED exposure, was achieved both in the presence (RPHy-LYZ) and in the absence (RPHy) of LYZ, producing potential cell-embedding hydrogels for inducing either a transdifferentiation process or the cell death of triple-negative breast cancer cells. Our results suggest that these hydrogels may show good potential in anticancer treatment since the encapsulation of cancer cells within these systems could provide targeted and localized treatment while minimizing damage to healthy tissues. Among the potential advantages of using RPHy hydrogels that should be highlighted are the use of visible light instead of UV light, the possibility of precisely modulating the hydrogel mechanical properties that are known to greatly affect cell sensitivity to anticancer drugs [17,18], and the potential selective action on tumor cells since the hydrogel polymerization process could also lead to polymerization of ectopic LYZ found on cancer cells’ surface. Therefore, the results presented here open the way to a potential selective treatment with the RPL system of cancer cells on the basis of a high ectopic expression of LYZ and the presence of P in cancer cells.

## Materials and Methods

### LYZ polymerization by the RPL system

LYZ from chicken egg white (3 mg/ml) (Sigma-Aldrich, Milan, Italy) or bovine serum albumin (BSA) (3 mg/ml) (Sigma-Aldrich, Milan, Italy) were dissolved in 10 mM HEPES (Sigma-Aldrich, Milan, Italy) buffer (pH 8.5) and incubated with different concentrations of (−)-riboflavin (R) (Sigma-Aldrich, Milan, Italy) (50 and 500 μM R in double-distilled water) and P (Sigma-Aldrich, Milan, Italy) (25 μM and 10, 50, or 100 mM in 10 mM HEPES buffer pH 8.5). The optical density (OD) at 600 nm of LYZ solutions was evaluated after 24 h of incubation in the presence of visible light (LED exposure) at room temperature (21 °C). LYZ solutions and BSA solutions in the presence and in the absence of R (50 μM), P (25 μM), or RP (50 μM R and 25 μM P) were incubated for 3 or 24 h in the presence of light (L), and the samples were analyzed by sodium dodecyl sulfate–polyacrylamide gel electrophoresis (SDS-PAGE) using 15% or 7.5% (v/v) polyacrylamide gels.

### Spectrofluorimetric analyses

The fluorescence spectra of ThS (Sigma-Aldrich, Milan, Italy) (0.5 μg/ml) in 10 mM HEPES buffer (pH 8.5) were evaluated over time in the presence and in the absence of LYZ or BSA (1 mg/ml), 50 μM R, and 25 μM P and LED exposure at 25 °C. After 12 h of light exposure, the solutions were centrifuged for 30 min at 12,000 g at room temperature, and then the supernatants were recovered and analyzed by fluorescence spectroscopy. The spectra were recorded using an LS50 PerkinElmer spectrofluorometer using an excitation of 391 nm, a wavelength emission range of 400 to 600 nm, a slit of 3 nm, and a 500 nm/s scan speed, performing 2 accumulations. The intrinsic fluorescence of LYZ (0.2 mg/ml) at different concentrations of P (0, 25, 50, 98, and 122 μM) was assessed using an excitation of 280 nm and an emission range of 300 to 400 nm, a slit of 3 nm, a scan speed of 500 nm/s, and 2 accumulations.

### Cell culture production and characterization

#### Bi- and 3-dimensional cell cultures

Cell studies were performed on MDA-MB 231, normal human dermal fibroblasts (NHDFs) (Lonza, Basel, Switzerland), bone-marrow-derived mesenchymal stem cells (BMMSCs) (Gibco, StemPro, Life Technologies, Italy), and human cardiac Lin^−^ Sca1^+^ progenitor (cMSC) cell lines. cMSCs were extracted by auricular biopsies made during coronary artery bypass surgery from patients after signing a written consent form as previously described [19,20]. The cell lines were cultured at 37 °C, 5% CO_2_, in high-glucose Dulbecco’s modified Eagle medium (Gibco, Italy) containing 10% fetal bovine serum (v/v) (Gibco, Italy), 1% penicillin–streptomycin (w/v) (Sigma-Aldrich, Italy), and 2 mM l-glutamine solution (Gibco, Italy). For the 3D cell cultures, a polyethylene glycol (PEG)–fibrinogen hydrogel (PFHy) precursor solution was prepared according to a published protocol [18,19,21]. Briefly, the PFHy precursor solution was diluted with phosphate-buffered saline (PBS) to obtain a final concentration of 8 mg/ml fibrinogen, and 0.5% (v/v) PEGDA (10 kDa) was added. Cells were resuspended in the precursor solution at a cell density of 10^6^ cells/ml, and photopolymerization was performed by addition of 1% (v/v) Irgacure 2959 (Ciba Specialty Chemicals, Basel, Switzerland) and exposure to UV light (365 nm, 4 to 5 mW cm^−2^) for 5 min.

Bright-field microscopy of the cell cultures was performed using a Zeiss microscope (Primovert, Zeiss, Italy).

#### Cell viability assay and FACS analysis

The cell viability of 2-dimensional (2D) and 3D cell cultures was assessed after 24 and 48 h of treatment, respectively, by 4-[3-(4-lodophenyl)-2-(4-nitrophenyl)-2*H*-5-tetrazolio]-1,3-benzene disulfonate (WST-1) assay (Cell Proliferation Reagent WST-1, Roche, Mannheim, Germany) [22]. Cell treatments in 2D cultures were performed by seeding 2 × 10^4^ cells/cm^2^ and adding to the cell culture medium R (10, 50, or 100 μM) and P (25 or 100 μM) or RP (50 μM R and 25 μM P) 24 h after seeding. Three-dimensional cell cultures were treated with RP (50 μM R and 25 μM P) after 24 h of cell growth. The cells were cultured in normal and light conditions. After each treatment, the medium was replaced with fresh high-glucose Dulbecco’s modified Eagle medium without phenol red (Gibco, Italy) containing tetrazolium salt WST-1 (5% v/v) and then incubated for 4 h at 37 °C and 5% CO_2_. The absorbance of the medium was evaluated at a wavelength of 450 nm using the microplate reader iMark Microplate Absorbance Reader (Bio-Rad).

Cell cycle distribution analysis was performed by flow cytometry (fluorescence-activated cell sorting [FACS] analysis) after 3 or 24 h of L (light) or RPL treatment. Briefly, cells (about 0.5 × 10^6^) were harvested and stained with 50 μg/ml propidium iodide (PI) (Sigma-Aldrich, Italy) in PBS buffer using 0.1% Triton X-100 and 1 mg/ml sodium citrate. Subsequently, the samples were incubated for 30 min at 4 °C and analyzed using a FACSCalibur flow cytometer (Beckton Dickinson, San Jose, CA, USA) and the percentage of cells in each phase of cell cycle was evaluated according to Nicoletti et al. [23]. The data obtained were then elaborated with the WinMDI software.

#### NMR spectroscopy for cellular polar phosphatidylcholine metabolite quantification

Deuterated reagents (methanol [CD_3_OD] and chloroform [CDCl_3_]) and deuterium oxide (D_2_O) (Cambridge Isotope Laboratories, Inc.) and 3-(trimethylsilyl)propionic-2,2,3,3-*d*_4_ acid sodium salt (TSP) (Merck & Co, Montreal, Canada) for nuclear magnetic resonance (NMR) analyses were used.

The human breast cancer cell line MDA-MB 231 (triple negative: ER^−^, PgR^−^, and HER2^−^) and the nontumorigenic immortalized HMEC line MCF-10A were supplied by the American Type Culture Collection (Manassas, VA, USA). The cells were cultured, as previously described [24]; cell pellets were resuspended in ice-cold extraction solvents (methanol/chloroform/water [1:1:1]) and vigorously vortexed for the intracellular metabolome. At least 24 h later, polar and lipid phases were separated by centrifugation at 20,000 g at 4 °C for 30 min. Next, the polar methanol/water phase containing water-soluble cellular metabolites was lyophilized using a rotary evaporator (Savant RTV 4104 freeze dryer), while the organic phase (lipid phase) was collected in a tube, and chloroform was evaporated under nitrogen gas flow. Both phases of extract cells were stored at −20 °C. Afterward, the samples were centrifuged at 14,000 g for 30 min and the supernatant obtained was then freeze-dried in a Savant RTV 4104 freeze dryer. The aqueous fractions from cells and extracellular media were reconstituted in 700 μl of D_2_O using TSP (0.1 mM) as NMR internal standards. High-resolution ^1^H NMR analysis was performed at 25° C at 400 MHz (9.4-T Bruker AVANCE spectrometer, Karlsruhe, Germany, Europe) on aqueous and organic cell extracts using acquisition pulses, water presaturation, data processing, and peak area deconvolution. The absolute quantification of aqueous metabolites, determined by comparing the integral of each metabolite to the integral of the reference standard TSP and corrected by respective proton numbers for metabolite and TSP, was expressed as nanomoles/10^6^ cells (nmol/10^6^) [25].

#### Fluorescence microscopy and ThS staining

Fluorescence analysis of 2D MDA-MB 231 cells and of 3D cell samples (MDAPF, MDA-MB 231 cells embedded in PFHy) was performed using live-cell fluorescent staining of the nuclei with Hoechst 33342 (Sigma-Aldrich, Italy) for 4 h and then fixing the samples with 4% (w/v) paraformaldehyde (PFA) in PBS for 30 min at room temperature. Permeabilization of the cells was performed using 0.3% (v/v) Triton X-100 solution for 5 min, and after washing in PBS, the samples were incubated in a blocking buffer (10% [v/v] fetal bovine serum, 0.1% [v/v] Triton X-100, and 1% [w/v] glycine in PBS) overnight at 4 °C.

Immunofluorescence microscopy was performed using the primary antibody mouse Ab-LYZ (lysozyme) (Proteintech, Manchester, UK) and the secondary antibody Alexa Fluor 546 fluorochrome-conjugated mouse Ab (Sigma-Aldrich, Milan, Italy).

ThS staining of 2D cell cultures was performed on the culture medium of MDA-MB 231 as follows: the medium was recovered and centrifuged for 3 min at 2,000 g, and the pellet was fixed on a slide with 4% (w/v) PFA and stained with ThS (1% [w/v] dissolved in H_2_O_dd_) [26]. After washing in PBS, the samples were analyzed by fluorescence and confocal microscopy (*λ*_ex_ 430 nm and *λ*_em_ 540 nm) using a Zeiss Axio Observer 7 fluorescence microscope and a Stellaris Leica microscope platform, respectively.

ThS staining was also performed on 2D seeded MDA-MB 231 previously fixed with 4% PFA and permeabilized and afterward nuclei staining with Hoechst 33342 or with PI. Samples were analyzed by confocal microscopy using a Stellaris Leica microscope platform.

After blocking, 3D MDAPF samples were incubated for 2 h at room temperature with 1:200 v/v phalloidin-conjugated Alexa Fluor 660 (Thermo Fisher Scientific, Invitrogen, USA) in PBS with 1% albumin and 20 mM Gly solution, and the samples were analyzed by confocal microscopy.

#### Protein extraction and western blotting analysis

Protein extraction from MDA-MB 231 and BMMSCs was performed using radioimmunoprecipitation assay buffer (100 μl) containing a protease inhibitor cocktail (Sigma-Aldrich, Milan, Italy) and pervanadate (Sigma-Aldrich, Italy) as a phosphatase inhibitor. After incubation for 90 min in ice, lysates were centrifuged for 10 min at 8,000 g at 4 °C. Bicinchoninic acid protein assay (Sigma-Aldrich, Milan, Italy) was used to determine the protein content, and SDS-PAGE of cell extracts (30 μg of protein) was performed using 15% polyacrylamide gel. For electroblotting, polyvinylidene fluoride membranes (Sigma-Aldrich, Italy) were used, blocked, and probed with the primary monoclonal antibodies mouse Ab-LYZ (Proteintech, Manchester, UK) and rabbit Ab-cyclin D1 (Cell Signaling Technology, USA). Immunoblots were next incubated with the mouse and rabbit secondary antibodies (dilution 1:3,000) (Cell Signaling Technology, USA) for 4 h at room temperature. Immunoblots with Ab-β-tubulin–horseradish peroxidase conjugates (Abcam, Milan, Italy) were also probed for controlling the protein loading. A SuperSignal West Pico kit (Thermo Scientific, USA) was used to visualize signals, followed by exposure to a FluorChem Imaging system (Alpha Innotech Corporation-Analitica De Mori, Milan, Italy).

### RPHy and RPHy-LYZ synthesis and analysis of antimicrobial properties

The RP hydrogel (RPHy) was obtained by preparing the following precursor solution in 10 mM HEPES buffer, at pH 8.5: 20% PEGDA 575 (v/v) (Sigma-Aldrich, Milan, Italy), 5 or 50 mM P (Sigma-Aldrich, Milan, Italy), and 0.5 mM R (Sigma-Aldrich, Milan, Italy). The RPLyz hydrogel (RPHy-LYZ) was obtained by the addition of 5 mg/ml of LYZ. The precursor solutions underwent sol–gel transition when exposed to an LED for 5 to 15 min depending on the considered volume (5 to 100 μl).

The antimicrobial activity of both RPHy and RPHy-LYZ was tested using the ampicillin-resistant *Escherichia coli* (*E. coli*^AmpR^) BL21 strain grown in solution with Luria–Bertani (LB) medium with 100 μg/ml of ampicillin. The hydrogels were placed in the LB medium seeded with the bacterial overnight culture and incubated at 37 °C. The cultures in LB solution were followed on time (24 h) monitoring the OD at 600 nm.

### Three-dimensional MDA-RPHy culture and characterization

MDA-MB 231 cells were resuspended in RPHy precursor solutions at a cell density of 10^4^ cells/μl, and microspheres of 5 μl were dropped on a nanostructured superhydrophobic surface of polydimethylsiloxane fabricated here using the procedure previously described [21]. The hydrogel polymerization was obtained by exposing the cell-loaded microspheres to an LED for 5 min.

Cell extraction by the MDARPHy spheres was performed using the following protocol: the spheres were incubated with trypsin–EDTA solution (Sigma-Aldrich, Milan, Italy) for 5 min, and after centrifugation at 2,000 g for 3 min, the centrifuged solution was placed in a multiwell plate. After cell adhesion, Alizarin Red S (Sigma-Aldrich, Italy) staining of the MDA-MB 231 cells for the identification of calcium-rich deposits was performed by fixing the cells with 4% PFA and staining with Alizarin Red solution, pH 4.1, for 10 min and washing with H_2_O_dd_, according to the specific protocol. The staining was assessed by optical microscopy using a Zeiss microscope (Primovert, Zeiss, Italy).

MDARPHy-LYZ 3D systems were produced using a cell density of 1.75 × 10^3^ cells/μl and 5 mg/ml of LYZ in the RPHy precursor solution, using a syringe as a mold and photopolymerization by 15 min of LED exposure.

### Protein structure and interaction prediction

Three-dimensional structural models of chicken-LYZC protein (LYZ) with R and P were obtained using AlphaFold 2 protein folding [26] and DiffDock-L [27] using the protein sequence of human LYZ-C (P61626).

### Statistical analysis

GraphPad Prism version 8.0 for Windows (GraphPad Software, San Diego, CA, USA) was used for the statistical analysis. Data obtained from 3 or more experimental independent biological replicates were quantified and analyzed for each variable using a *t* test or one-way analysis of variance (ANOVA) test followed by Dunnett’s multiple-comparisons test. For the NMR analyses, the one-way ANOVA test was followed by Bonferroni’s multiple-comparisons test. *P* value < 0.05 was considered statistically significant. Data are presented as mean ± standard deviation (SD).

## Results

### RPL sensor system identification and effect evaluation

#### Effects of the RPL system on proteins

**Fig. 1.**
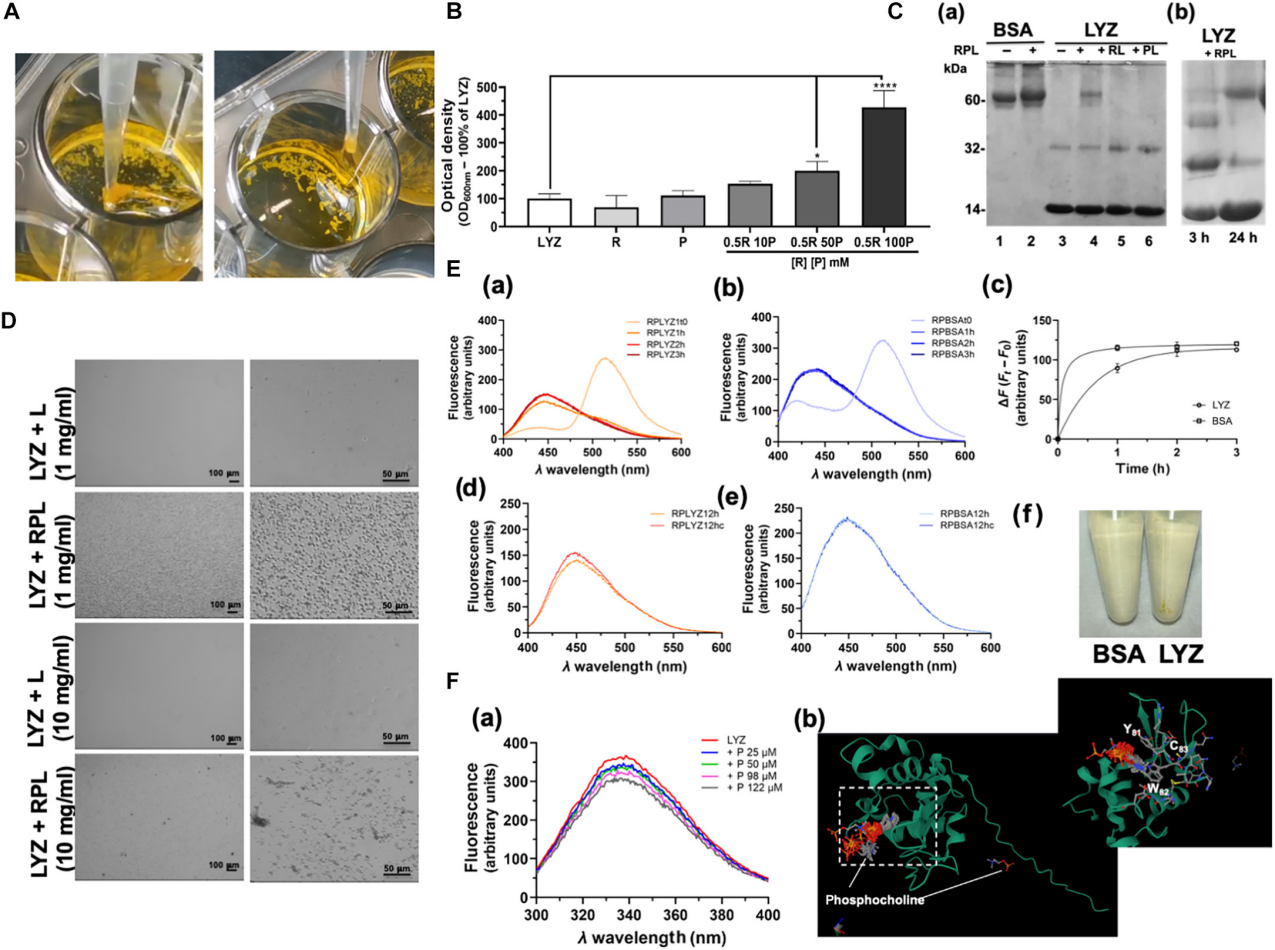
The riboflavin–phosphocholine–light (RPL) system induces lysozyme (LYZ) photopolymerization. (A) Digital images of LYZ’s gel-like aggregate formation after 24 h of RPL treatment (0.5 mM riboflavin [R] and 100 mM phosphocholine [P]) of the LYZ solutions (3 mg/ml LYZ). (B) Optical density at 600 nm of LYZ solutions incubated for 24 h with R (0.5 mM), with P (10 mM), or with RP (0.5 mM R and 10, 50, or 100 mM P) and exposed to a light-emitting diode (LED). (C) (a) Sodium dodecyl sulfate–polyacrylamide gel electrophoresis (SDS-PAGE) after 24 h of LED exposure of bovine serum albumin (BSA) solutions (3 mg/ml) (lanes 1 and 2) in the absence and in the presence of RPL (50 μM R and 25 μM P) and of LYZ solutions (3 mg/ml) (lanes 3 to 6): 3, LYZ; 4, + RPL; 5, + RL (50 μM); 6, + PL (25 μM). (b) SDS-PAGE of LYZ + RPL after 3 and 24 h of light exposure. (D) Bright-field micrographs of LYZ solutions (1 and 10 mg/ml) in the presence and in the absence of RP after 12 h of LED exposure. (E) Fluorescence spectra (*λ*_ex_ = 391 nm) of thioflavin S (ThS) solutions in the presence of (a) LYZ and (b) BSA (1 mg/ml of LYZ or BSA) with RP at time 0 and after 1, 2, and 3 h of LED exposure. (c) Fluorescence variation (Δ*F*) over time at 446 nm of ThS in LYZ and BSA solutions with RPL. Fluorescence spectra of ThS in the presence of (d) LYZ + RPL or (e) BSA + RPL after 12 h, recorded before (RPLYZ12h and RPBSA12h) and after pellet removal (RPLYZ12hc and RPBSA12hc). (f) Digital images of LYZ + RPL and BSA + RPL after centrifugation. (F) (a) Fluorescence spectra (*λ*_ex_ = 280 nm) of LYZ solution in the presence of increasing concentrations of P (0, 25, 50, 98, and 122 μM). (b) Three-dimensional (3D) LYZ structure with the predicted interaction sites of P obtained using the AlphaFold 2 program [26,27]. Experiments were performed in 3 or 6 replicates. The error bar indicates the SD. *P value ≤ 0.05; ****P value ≤ 0.0001. Scale bars are 100 and 50 μm.

The possibility of using the RPL system for the first time as a polymerizing system was evaluated here, considering that the triplet-excited R molecule can accept an electron, while P could act as a co-initiator. P can become a radical cation and facilitate electron transfer by acting as a cross-linker, thus initiating the radical photopolymerization process. The polymerization ability of the RPL system was initially tested on LYZ, as an amino acid polymer. LYZ was incubated in the presence and in the absence of R (0.5 mM) and P (10 mM) and combining RP at increasing concentrations of P (ranging from 10 to 100 mM); then, the solutions were exposed to visible light (LED) for 24 h at 25 °C. An abundant formation of polymeric LYZ gel-like aggregates was found in the samples treated with the RPL system (Fig. 1A). The protein aggregation process occurring with the addition of R and P was quantitatively assessed through spectrophotometric analysis, evaluating the increase in OD at 600 nm (Fig. 1B). To assess whether the LYZ aggregation resulted from a polymerization process, possibly due to cross-link formation, SDS-PAGE was performed. After 24 h of light treatment of LYZ solutions, the presence of high-molecular-weight species was detected only in the samples treated with the combination of R and P (Fig. 1C (a)). In particular, there is evidence of dimeric forms at about 30 kDa and tetrameric forms at approximately 60 kDa. Evaluating the oligomer formation over time, it was observed that the dimeric, trimeric, and tetrameric species started forming as early as 3 h, increasing after 24 h due to the formation of additional cross-links (Fig. 1C (b)). The same experiments performed with BSA did not show the formation of oligomeric species (see Fig. 1C (a)).

Microscopy and fluorescence studies were also performed to better evaluate the photopolymerization process of LYZ. In Fig. 1D are shown the micrographs of the LYZ solutions in the presence and in the absence of RP after 12 h of LED exposure, and a high protein aggregation in the presence of RP was observed. The formation of LYZ fibrils was also evaluated by fluorescence spectroscopy monitoring the fluorescence changes of the fluorophore ThS (Fig. 1E (a) and (b)). The presence of R in the solution led us to assess the ThS fluorescence at 446 nm after excitation at 391 nm. The spectra of the LYZ and BSA solutions with RP and ThS after LED exposure are shown in Fig. 1E, demonstrating that after 1 h of LED exposure, both protein solutions showed a high increase in fluorescence at 446 nm, which was related to the disappearance of the maximum at 515 nm due to the R species. Moreover, more hours of incubation in light conditions led to an increase in fluorescence at 446 nm of the LYZ solution, which was not detected in the case of the BSA solution, as shown in Fig. 1E (b) and (c). This increase can be related to the formation of LYZ fibrils due to the photopolymerization process induced by the RPL system. After overnight LED exposure, fluorescence quenching was observable due to an inner filtering effect of the LYZ polymeric species that was abolished by centrifugation and removal of the pellet (Fig. 1E (d) and (f)). Neither fluorescence changes nor precipitates were observed in the case of BSA at the same conditions (Fig. 1E (e) and (f)). These data demonstrate a selective photopolymerization effect of the RPL system on specific proteins, such as LYZ.

To better study the specificity of the RPL system, the interaction between LYZ and P was also evaluated by fluorescence spectroscopy, observing the quenching of the intrinsic fluorescence of LYZ in the presence of P in a concentration-dependent manner (Fig. 1F (a)). In Fig. 1F (b) is shown the 3D LYZ structure with the predicted interaction sites of P obtained using AlphaFold 2 program (AlphaFold 2 protein folding and DiffDock-L) [27] (see Fig. S1). In this 3D structural model of human LYZC interacting with P are present 2 residues, Y81 and W82, whose fluorescence could be affected by the presence of P. Therefore, this prediction of the molecular LYZ–P interaction is in agreement with the observed intrinsic fluorescence changes of LYZ in dependence of the P concentration, observed in chicken-LYZC protein, used for our fluorescence experiments, where in the same position the W80 and W81 residues are present.

#### Effects of the RPL system on different cell lines

**Fig. 2.**
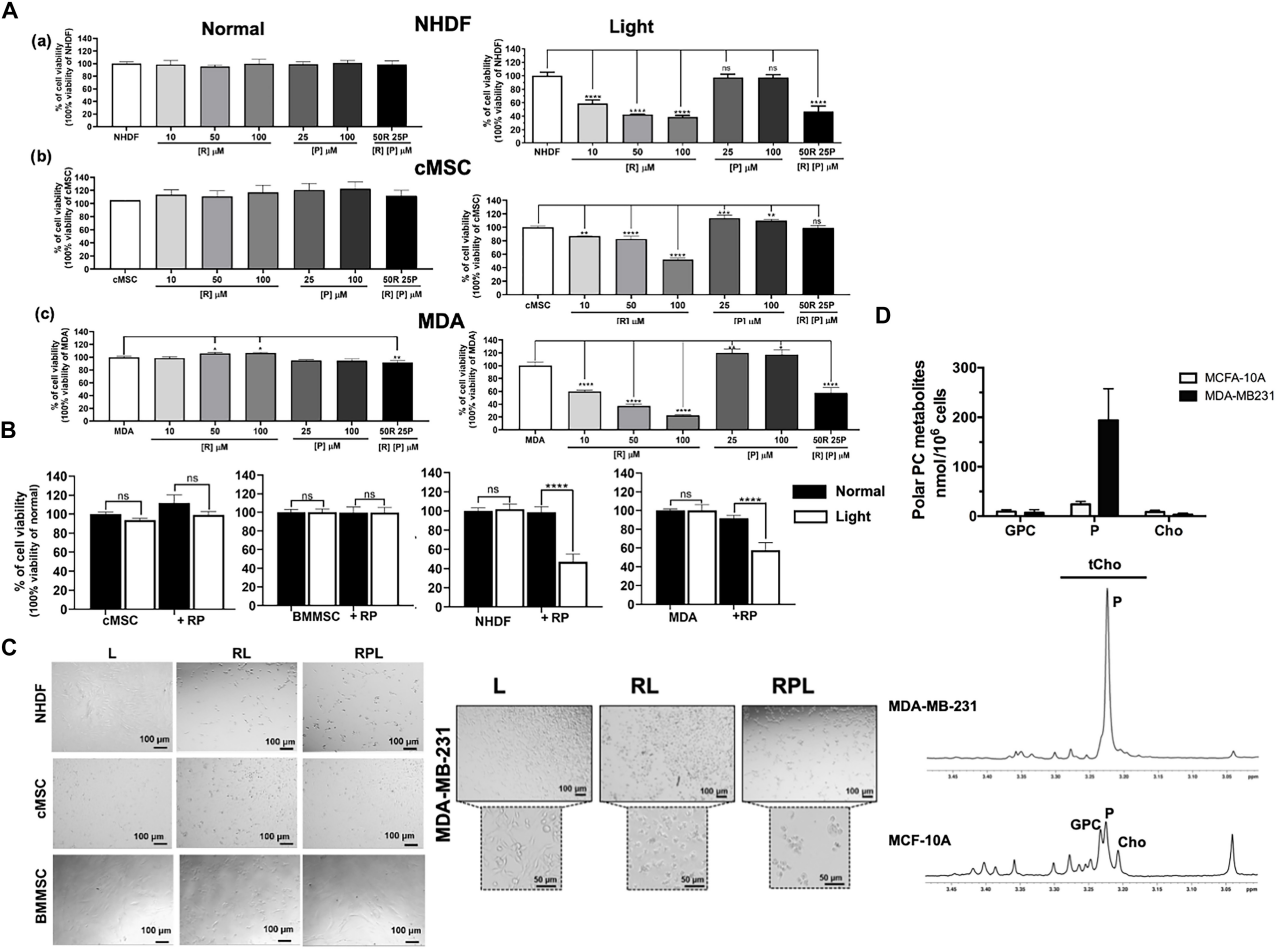
Effects of the RPL system on fibroblasts and tumor and mesenchymal stem cells. (A) WST-1 cell viability assay of (a) normal human dermal fibroblasts (NHDFs), (b) human cardiac Lin^−^ Sca1^+^ progenitor (cMSCs), and (c) MDA-MB 231 cells (MDA) treated with 10, 50, or 100 μM R; with 25 or 100 μM P; or with RP (50 μM R and 25 μM P). Cell viability was assessed after 24 h of treatment, keeping the samples in standard conditions (normal) of cell growth or exposed to LED. (B) Comparison of the cell viability of cMSCs, BMMSCs, NHDFs, and MDA treated for 24 h with or without RP (50 μM R and 25 μM P) in standard conditions of cell growth and under LED exposure. (C) Bright-field micrographs of NHDFs, cMSCs, BMMSCs, and MDA-MB 231 exposed to light for 24 h without any treatment (L) and with the addition of R (50 μM) (RL) and of RP (50 μM R and 25 μM P) (RPL). (D) Expanded ^1^H nuclear magnetic resonance (NMR) spectral profiles (9.4 T) of the total choline (tCho) resonances in aqueous extracts of nontumoral MCF-10A and MDA-MB 231 cancer cells and below the absolute concentrations (nmol/10^6^ cells) of their phosphatidylcholine (PC) metabolites determined by ^1^H NMR spectra. Experiments were performed as 3 or 6 biological replicates. The error bar indicates SD. One-way analysis of variance (ANOVA) tests were performed for the statistical analysis using multiple-group comparison test with Dunnet correction. *P value ≤ 0.05; **P value ≤ 0.01; ***P value ≤ 0.0005; ****P value ≤ 0.0001. Scale bars are 100 and 50 μm. ns, not significant; GPC, glycerophosphocholine; Cho, free choline.

**Fig. 3.**
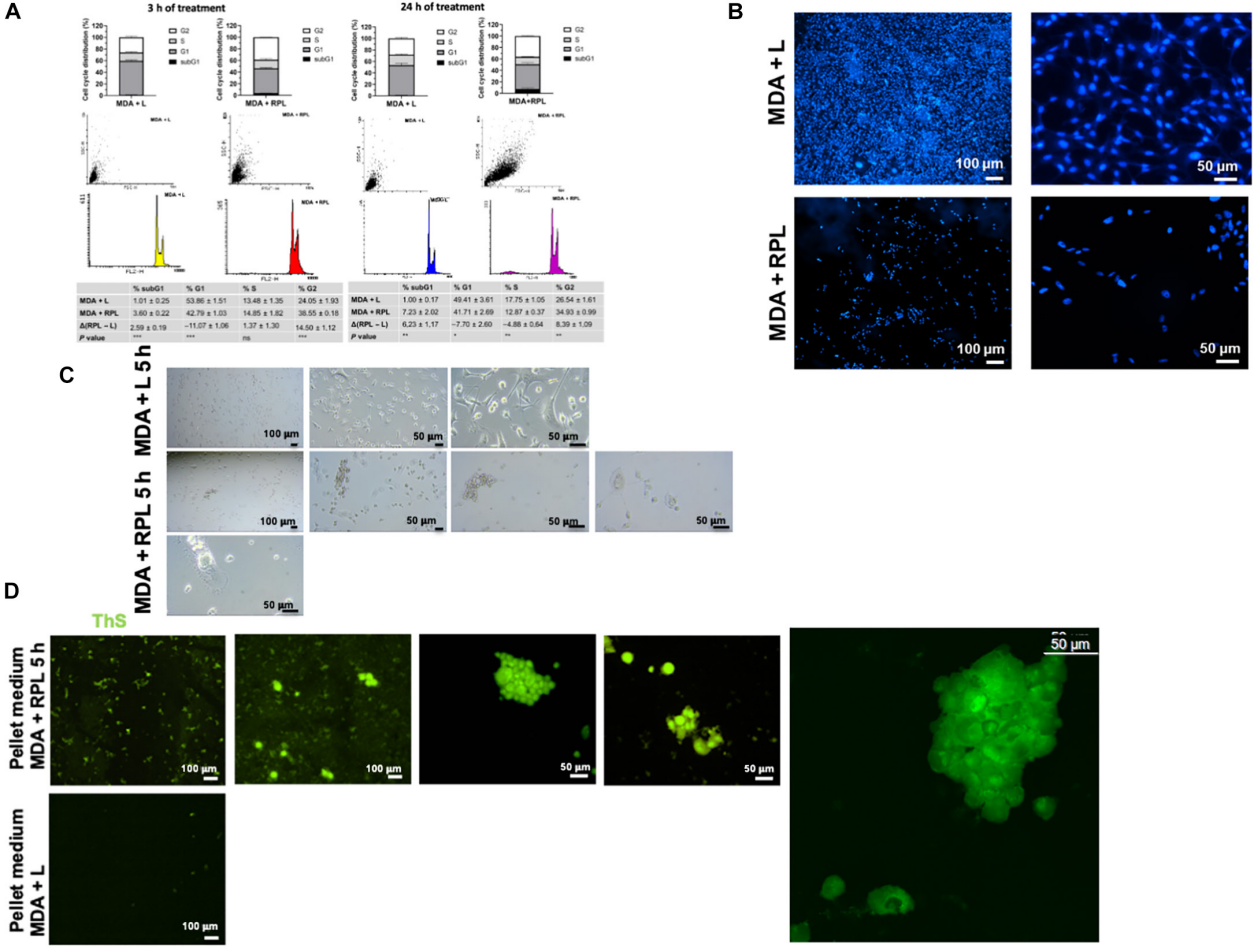
Effects of the RPL system on the cell cycle, morphology, and aggregation of MDA-MB 231 breast cancer cells. (A) FACS analyses of the MDA-MB 231 cells (MDA) cultured with LED exposure for 3 and 24 h in the presence (+ RPL) or in the absence (+ L) of RP (50 μM R and 25 μM P) as control; (B) fluorescence micrographs of MDA-MB 231 cells after 24 h of cell growth with LED in the presence or in the absence of RP, where the nuclei were stained with Hoechst 33342 (nuclei staining of live cells); (C) bright-field micrographs of MDA-MB 231 cells after LED exposure for 5 h with RP. After treatment, the medium was centrifuged, and in (D), there are the micrographs of the pellet of the medium stained with ThS (*λ*_ex_ = 430 nm and *λ*_em_ = 540 nm, magnification [Mag.] ×5 and ×20). Experiments were performed as 3 biological replicates. The error bar indicates SD. *P value ≤ 0.05; **P value ≤ 0.01; ***P value ≤ 0.0005; ****P value ≤ 0.0001. Scale bars are 100 and 50 μm.

The effects of the RPL system on different cell types were evaluated here. In Fig. 2A are shown the effects of the LED exposure (light) on the cell viability of NHDF, mesenchymal stem cell (cMSC and BMMSC), and breast cancer MDA-MB 231 cell lines using different concentrations of R and P. In standard conditions (normal) of cell growth, the addition of R and/or P did not induce any significant change in both normal dermal fibroblasts and mesenchymal stem cells, while an 8.4% (SD, 2.0%) decrease in cell viability in the cancer cell line was observed in the case of RP treatment. In contrast, the treatment with the RPL system induces significant decreases of 53% (SD, 8.2%) and 43% (SD, 8.0%) in the cell viability of the fibroblasts and breast cancer cells, respectively. The treatment with R in light conditions also induced a decrease in cell viability in a concentration-dependent manner. At a concentration of 50 μM R in the medium, the cell viability of cMSCs was 82.8% (SD, 3.6%), while the cell viability values of NHDFs and MDA-MB 231 cells were 42.5% (SD, 0.4%) and 37.3% (SD, 3.2%), respectively. In Fig. 2B are summarized the effects on cell viability of RP treatment for 24 h in the presence and in the absence of light. In agreement, also in the case of MSCs derived from bone marrow (BMMSCs), the RPL system did not induce significant changes in cell viability. In Fig. 2C are shown the micrographs of NHDFs, cMSCs, BMMSCs, and MDA-MB 231 cells after 24 h of treatment with the RL and RPL systems, where the effects of treatments on the cell morphology and viability of stem cells, fibroblasts, and cancer cells are visible. These differences were probably due to a different response to oxidative stress induced by radicals’ formation of R and to the presence of the endogenous P in the different cell types. One-dimensional ^1^H NMR analyses of aqueous extracts revealed a more intense total choline (tCho) containing metabolites’ resonance in MDA-MB 231 breast cancer cells compared to that in normal MCF-10A cells. The analysis of expanded tCho spectral profiles showed a significant increase in the P signal in breast cancer cells as compared to that in nontumoral MCF-10A (Fig. 2D), while the other polar phosphatidylcholine metabolites such as glycerophosphocholine and free choline did not change significantly. Abnormal phosphatidylcholine metabolism is a common feature in breast, ovary, and prostate cancer cell lines, with consequent alterations in the levels of NMR-detectable compounds, such as P and tCho. In particular, we found an increase of average 5-fold of the tCho level (tCho = 204.10 nmol/1 × 10^6^ cells) in MDA-MB 231 cells as compared to that in nontumoral MCF-10A cells (tCho = 41.6 nmol/1 × 10^6^ cells). A similar increase in tCho and P content was already observed in MDA-MB 231 as compared to normal human fibroblasts (tCho = 13.6 nmol/1 × 10^6^ cells) [28]. In those experimental models, spectral changes did not correlate with cell doubling times; thus, these signals have been previously proposed as fingerprints of tumor progression and/or endpoints of therapeutic treatment. The effects of the RPL system on the MDA-MB 231 cell line were also assessed by FACS (Fig. 3A), observing a significant increase in the subG1 and G2 cell populations over time (3 and 24 h), with respect to the control, only in the presence of light. In Fig. 3B are shown the fluorescence micrographs of the cells with and without RPL treatment, where the nuclei were stained with Hoechst 33342 (staining of the nuclei of live cells), and a high decrease in stained nuclei is visible. In Fig. 3C are also shown the bright-field micrographs of the cells after 5 h of RPL treatment, where the induction of cell aggregation/fusion processes is visible. To further investigate this cell–cell aggregation, the cell medium was recovered after 5 h of RPL treatment and the pellet was analyzed by fluorescence microscopy using ThS as fluorescent dye. In Fig. 3D are shown the micrographs of the pellet obtained after 5 h of treatment with the RPL system (+ RPL) and only after LED exposure as control (+ L). The RPL system induces detachment of the cells from the tissue culture plate with the formation of cellular aggregates well visible with ThS staining.

**Fig. 4.**
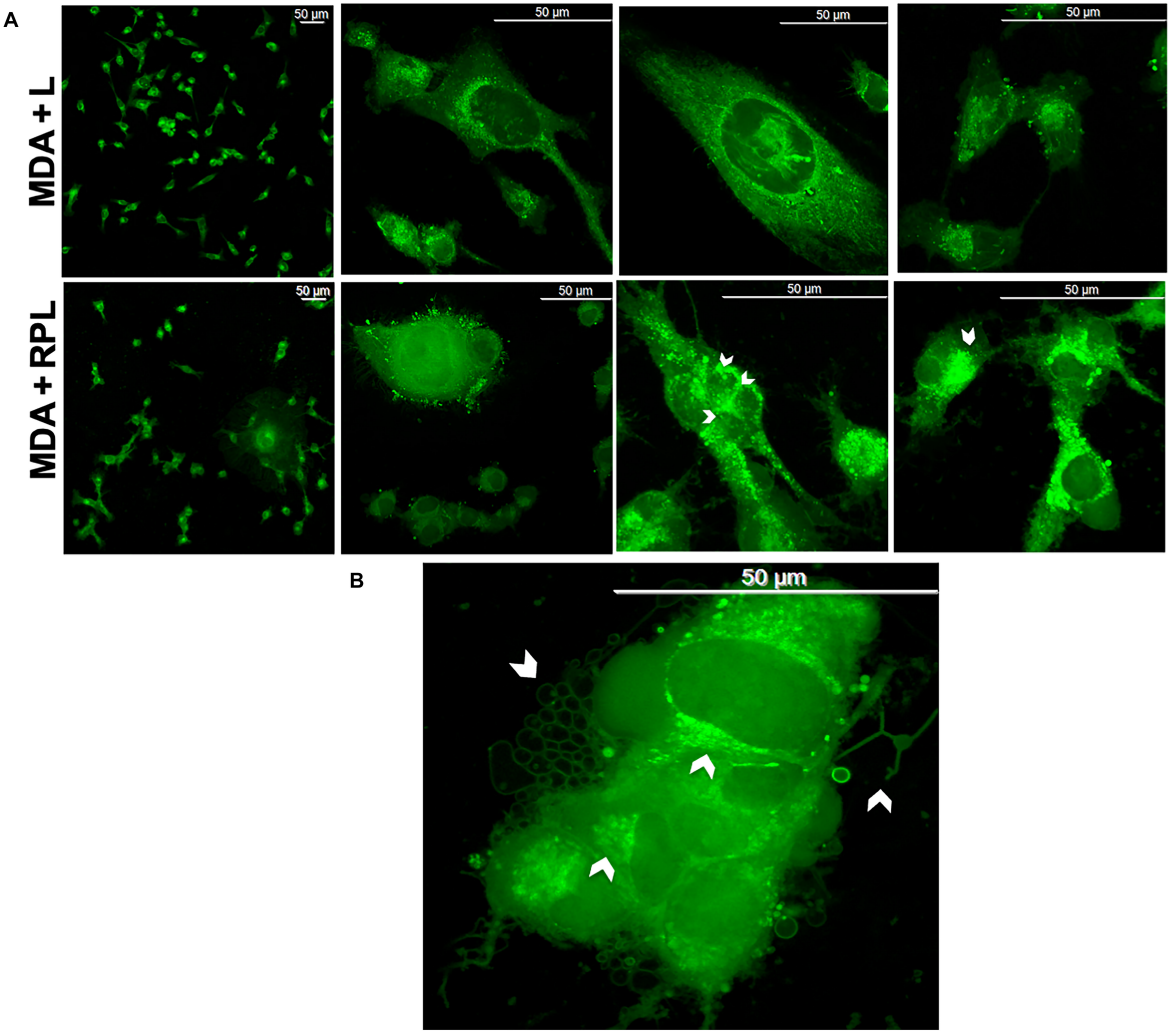
Effects of the RPL system on MDA-MB 231 cell aggregation. (A) Confocal fluorescence micrographs of MDA-MB 231 cells after 5 h of light treatment without (MDA + L) and with RP (50 μM R and 25 μM P) (MDA + RPL) added in the medium, stained with ThS, recorded using *λ*_ex_ = 430 nm and *λ*_em_ = 540 nm (Mag. ×20 and ×40); (B) magnification of MDA + RPL obtained using Mag. ×40, 3.6 zoom. The arrows indicate protein polymerization and cellular aggregation points. Scale bars are 50 μm.

After treatment, the cells were also analyzed by confocal fluorescence microscopy, and in Fig. 4 are shown the micrographs obtained after ThS staining (Fig. 4A and B), recording the ThS fluorescence at 540 nm. The cellular aggregates and reticulated extracellular structures, highlighted with arrows in Fig. 4A and B, are mainly present in the RPL-treated cells. Moreover, an increased fluorescence of ThS was visible in the cell–cell junction, indicating the possible formation of cell aggregation induced by protein–protein cross-links due to the RPL photopolymerization process.

**Fig. 5.**
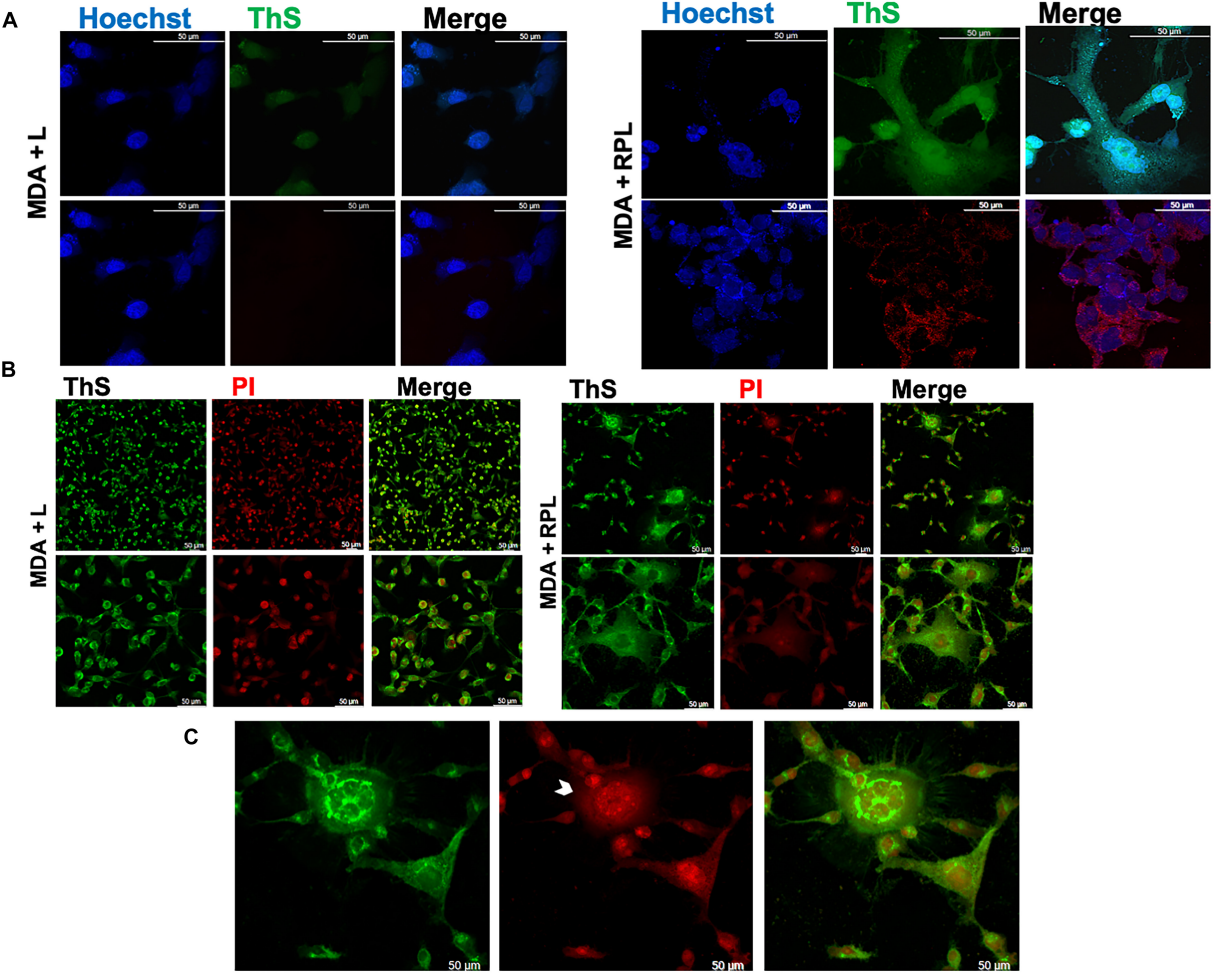
Confocal fluorescence micrographs of MDA-MB 231 cells treated with the RPL system. (A) Confocal micrographs of MDA + L and MDA + RPL where the nuclei were stained with Hoechst 33342 and then the cells were stained with ThS, recorded using *λ*_ex_ = 430 nm and *λ*_em_ = 540 nm (green) and *λ*_ex_ = 579 nm and *λ*_em_ = 603 nm (red) (Mag. ×40); (B) MDA + L and MDA + RPL where the nuclei were stained with propidium iodide (PI) recorded using *λ*_ex_ = 535 nm and *λ*_em_ = 615 nm and then the cells were stained with ThS, recorded using *λ*_ex_ = 430 nm and *λ*_em_ = 540 nm (Mag. ×20 and ×40); (C) magnification (×20, 2.2 zoom) of MDA + RPL stained with PI and ThS. Scale bars are 50 μm.

In Fig. 5A are shown the micrographs obtained staining with both ThS and Hoechst 33342 in which the formation of cell–cell aggregation and the presence of extensions of the cells stained with ThS are visible. Moreover, in order to avoid fluorescence interferences between the 2 fluorophores due to overlapping of the excitation picks of Hoechst 33342 and ThS, the nuclei were stained with PI, and the micrographs are shown in Fig. 5B. No fragmentation of the nuclei was observable after 5 h of RPL treatment in the case of single cells, but in contrast, pyknosis was visible in the cell–cell aggregates of the treated cells (Fig. 5C). Therefore, these results suggest that programmed cell death was induced by the cell–cell cross-linking induced by RPL treatment.

**Fig. 6.**
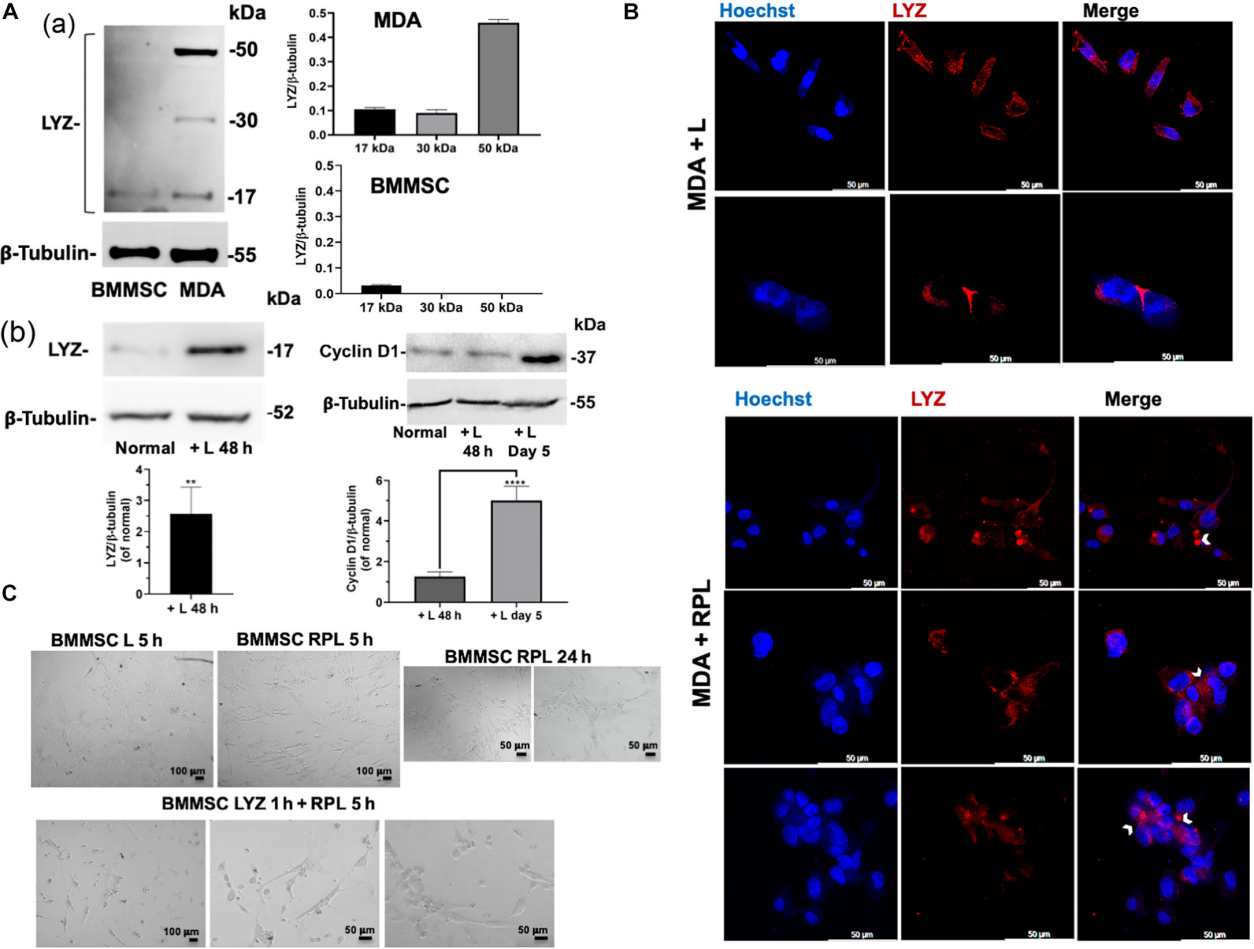
LYZ expression in different cells and localization in RPL-treated cells. (A) Western blot analysis showing the following: (a) The LYZ expression of BMMSCs and MDA-MB 231 after cell growth in standard conditions (normal). (b) LYZ and cyclin D1 expression after 48 h and 5 d of cell growth in the presence (+ L) or in the absence (Normal) of LED exposure, respectively. (B) Confocal micrographs of MDA-MB 231 (+ RPL) and without (+ L) RPL treatment; nuclei are stained in blue with Hoechst 33342, and LYZ is stained in red with Ab-LYZ-Alexa Fluor 546. The arrows indicate the cellular aggregation points, where the presence of LYZ (in red) is visible. (C) Bright-field micrographs of the BMMSCs after 5 and 24 h of RPL treatment in the absence and in the presence of 1 mg/ml of LYZ added in the cell culture medium 1 h before the addition of RP. Experiments were performed as 3 biological replicates. The error bar indicates SD. Scale bars are 100 and 50 μm.

LYZ expression mediates the effects of the RPL system on different cell lines. The molecular mechanism of this cell–cell aggregation process induced by RPL treatment is probably due to a protein–protein photopolymerization process. Recently, a different expression of LYZ dependent on cell type has been demonstrated [14]. On the basis of our molecular results for LYZ, discussed above, we investigated the hypothesis that the LYZ present in the extracellular matrix can have a relevant role in the photopolymerization process of the cells induced by RPL treatment and that the different expression of LYZ in the cells, together with the endogenous P concentration, can be a discriminant factor of the cell death induced by the treatment. LYZ expression was evaluated in the cell lines that showed great differences in behavior to the treatment, MDA-MB 231 and BMMSC cell lines (Fig. 6A). The LYZ expression in the BMMSCs grown in standard conditions of cell growth (normal) was much lower than that in MDA-MB 231 cancer cells (Fig. 6A (a)), and in the latter was observed the presence of LYZ formed at higher molecular weights (dimeric and trimeric forms), not present in the BMMSCs. This result may suggest that LYZ, which is a protein able to polymerize in the presence of RPL, can play a role in the cell–cell aggregation induced by RPL treatment. In addition, the levels of LYZ expression in NHDFs were more similar to that in MDA-MB 231 cells (see Fig. S2), in accord with the observed effects on cell viability (Fig. 2A).

Moreover, the expressions of both LYZ and cyclin D1 were also increased respectively after 48 h and 5 d of cell growth of MDA-MB 231 cells in the presence of LED exposure (see Fig. 6A (b)), indicating not only a correlation between tumor progression and light exposure but also that the effects of the RPL treatment could be potentiated on these tumor cells. The presence of LYZ in MDA-MB 231 cells was also investigated by confocal microscopy, and in Fig. 6B are shown the micrographs of the cancer cells after RPL treatment. A high expression of LYZ was observable between the cells in the cellular aggregates obtained by RPL treatment, suggesting the possible formation of LYZ cross-linking between the cells. The effect of pretreatment with LYZ of BMMSCs on RPL treatment was also evaluated, culturing the cells in the presence of 1 mg/ml of LYZ in the cell culture medium for 1 h before RPL treatment. Very significant morphological effects were observed in the BMMSCs after 5 h of RPL treatment, including loss of cellular adhesion and formation of spheroidal cellular aggregates similar to those seen in MDA-MB 231 cells treated with RPL (Fig. 6C). These effects were not observed in the absence of LYZ, even after 24 h of RPL treatment (Fig. 6C). Moreover, the simple LYZ pretreatment did not induce any visible changes to the cells (data not shown). Therefore, LYZ pretreatment can increase the ectopic LYZ concentration of BMMSCs, sensitizing the cells to RPL treatment.

#### Effects of the RPL system on 3D cancer cell cultures

**Fig. 7.**
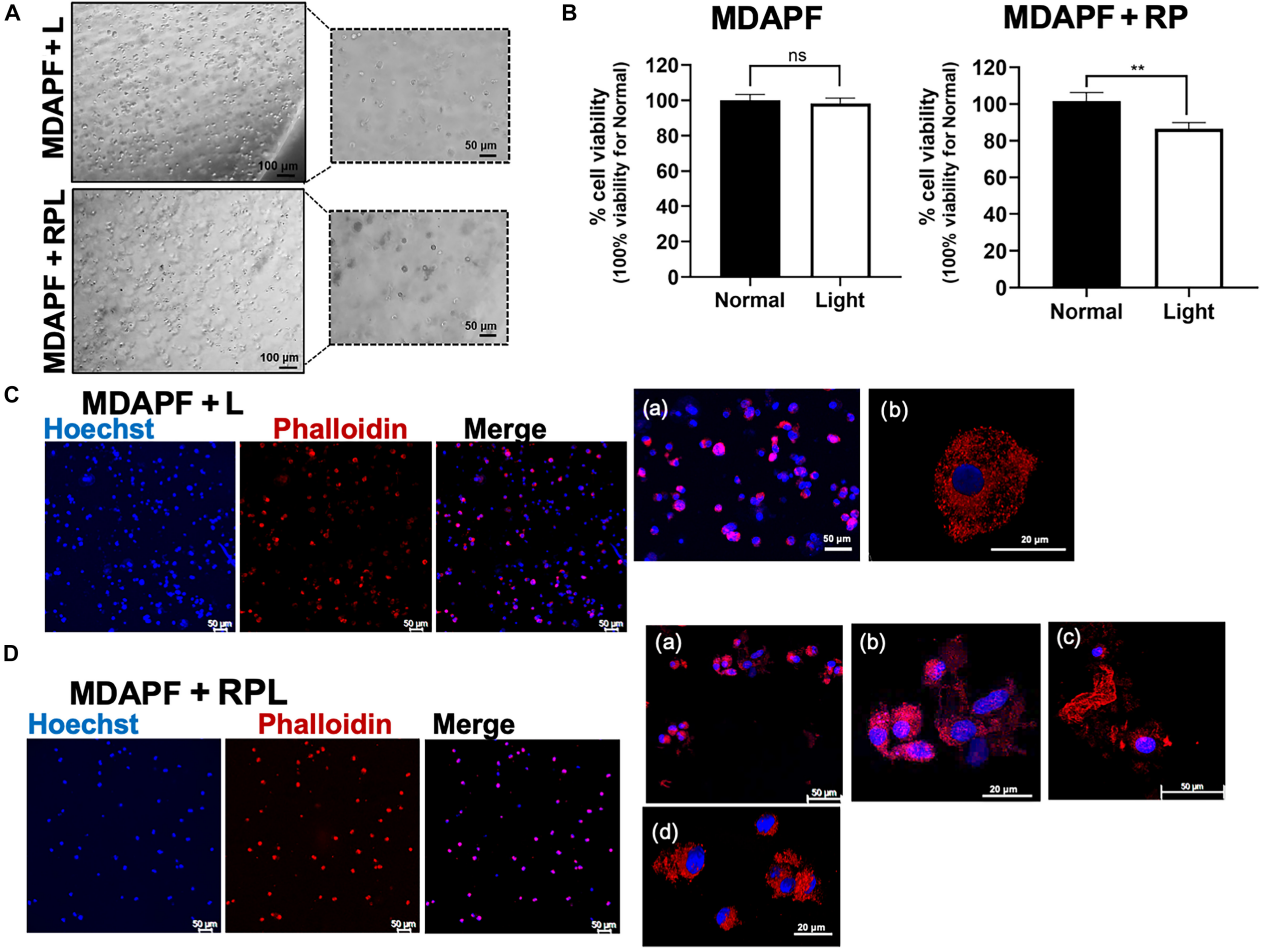
Effects of the RPL system on cell viability and morphology of breast cancer cells in 3D cultures. (A) Bright-field micrographs of MDA-MB 231 cells embedded in PFHy (MDAPF) at a cell density of 10^6^ cells/ml, exposed to light for 48 h without (MDAPF + L) and with (MDAPF + RPL) RPL treatment. (B) Cell viability of 3D cell cultures in standard conditions (Normal) or with exposure to LED light (Light) for 48 h without (MDAPF) and with (MDAPF + RP) RP treatment. Confocal micrographs of (C) MDAPF + L ((a and b) micrographs at higher magnifications) and (D) of MDAPF + RPL; nuclei are stained in blue with Hoechst 33342, and the cytoskeleton is stained in red using phalloidin-conjugated Alexa Fluor 660: (a to d) micrographs at higher magnifications. All experiments were performed as 3 biological replicates. The error bar indicates SD. **P value ≤ 0.01. Scale bars are 100 and 50 μm.

The effects of RPL treatment were also evaluated on 3D cell growth systems of tumor cells. Tumoroids of MDA-MB 231 cells were produced using PFHy as previously described [18,19,21]. The cells were embedded via photopolymerization in PFHy molds (5 mm diameter × 3 mm height), and cell growth was carried out for 48 h in the presence or in the absence of RPL treatment. In Fig. 7A are shown bright-field micrographs of the molds, and in Fig. 7B are shown their cell viabilities obtained by WST-1 assay. In agreement with the 2D cell culture, in standard conditions of cell growth, no changes in cell viability were observed either in the presence or in the absence of RP, but a statistically significant decrease in cell viability of 15.03% (SD, 2.9%) was observed in the presence of LED exposure. Moreover, the effects of the RPL treatment on cell morphology were also assessed using phalloidin staining of the cells, as shown in Fig. 7C and D. The presence of morphological changes and cellular aggregates of the cancer cells treated with RPL was clearly visible, as shown in Fig. 7D.

### RPL system for producing LED-photopolymerizable PEG hydrogels (RPHys)

#### RPHys synthesis and characterization

**Fig. 8.**
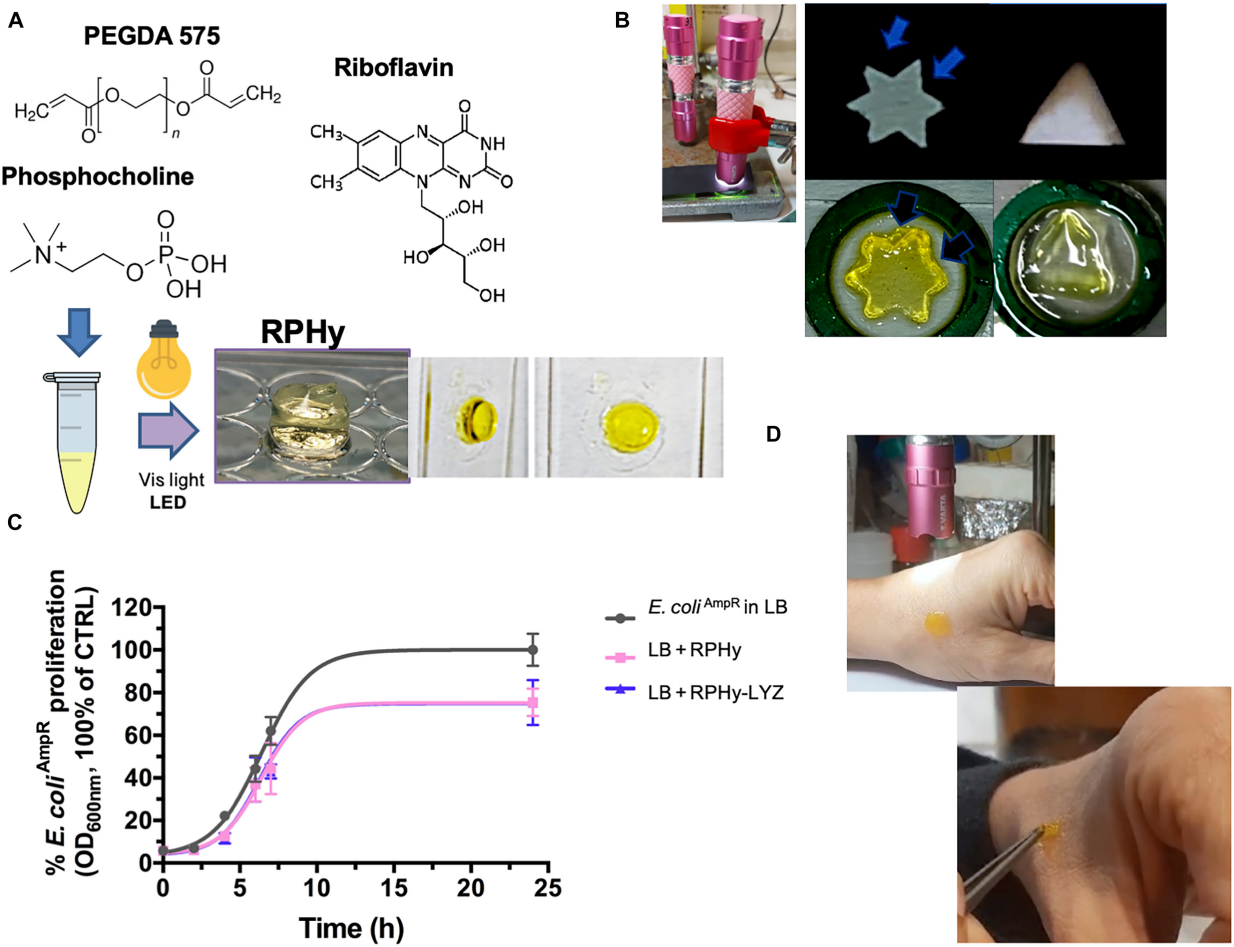
RPHys synthesis and characterization. (A) Molecular structures of RPHy components and schematic representation of the hydrogel preparation obtained from the precursor solution in 10 mM HEPES buffer, at pH 8.5, containing 20% polyethylene glycol diacrylate (PEGDA) 575 (v/v), 5 mM P, and 0.5 mM R, exposed to LED light. (B) Selective photopolymerization tests using masks with specific geometries. (C) Ampicillin-resistant *Escherichia coli* (*E. coli*^AmpR^) growth is obtained by OD_600nm_ over time. *E. coli* growth was performed in Luria–Bertani (LB) medium with 100 μg/ml ampicillin (black line) and in the presence of RPHy (pink line) or RPHy produced with the addition of 5 mg/ml of LYZ (RPHy-LYZ) (purple line). (D) Digital images representing tests of RPHy photopolymerization on skin (see Video S1). Experiments were performed as 3 independent replicates. The error bar indicates SD. Vis, visible.

Based on the ability of R and P to induce the formation of protein polymers in the presence of LED exposure, here, the possibility of achieving a similar process using PEGDA was also investigated. In particular, PEGDA 575 was chosen, considering its potential application in 3D printing. Several experiments were conducted using different concentrations of R, P, and PEGDA 575 along with different LED exposure times. This allowed for the optimization of the photopolymerization process, leading to the development of a protocol for the production of a hydrogel, as illustrated in Fig. 8A. Various compound percentages were evaluated, leading to the identification of an optimal composition (20% [v/v] PEGDA, 0.5 mM R, 5 mM P, and 10 min of LED exposure) to obtain a hydrogel with suitable strength and elasticity. This was achieved through LED photopolymerization without the use of nonnatural substances. A yellow-colored hydrogel was obtained due to the presence of R, but this color was reduced over time by light exposure. The hydrogel was used to produce 3D cell cultures, observing that the concentration of P influences the final properties of the hydrogel. In this context, 2 hydrogels prepared with different concentrations of P (5 and 50 mM) were also produced, revealing a direct correlation between the increase in P concentration and hydrogel stiffness (data not shown). The increase in stiffness is attributed to the formation of a greater number of internal cross-links during the photopolymerization phase induced by P. The feasibility of using the mixture as an “ink” for 3D printing with visible light was also assessed here by selective photopolymerization tests conducted using masks with specific geometries. In this photopolymerization process, visible light radiation was prevented from reaching the entire hydrogel by positioning a “mask”, demonstrating a selective photopolymerization of the precursor solution only in the areas not covered by the “mask” (Fig. 8B). Moreover, we added LYZ (5 mg/ml) to the hydrogel composition as the protein component of the hydrogel, obtaining RPLYZ hydrogel (RPHy-LYZ), without any difficulty in the photopolymerization process, thanks to the ability of LYZ to photopolymerize in the presence of RP. In contrast, not a good polymerization occurred in the presence of albumin at the used concentrations (data not shown). The antimicrobial activity of both the hydrogels RPHy and RPHy-LYZ was tested using the bacterial culture of the *E. coli*^AmpR^ BL21 strain, as shown in Fig. 8C. The presence in the bacterial culture of both hydrogels induced a decrease in bacterial growth, which was monitored by OD at 600 nm. Finally, we performed hydrogel photopolymerization directly on human skin, demonstrating that it can occur directly on the skin (see Fig. 8D and Video S1).

#### RPHys for cancer cell embedding

**Fig. 9.**
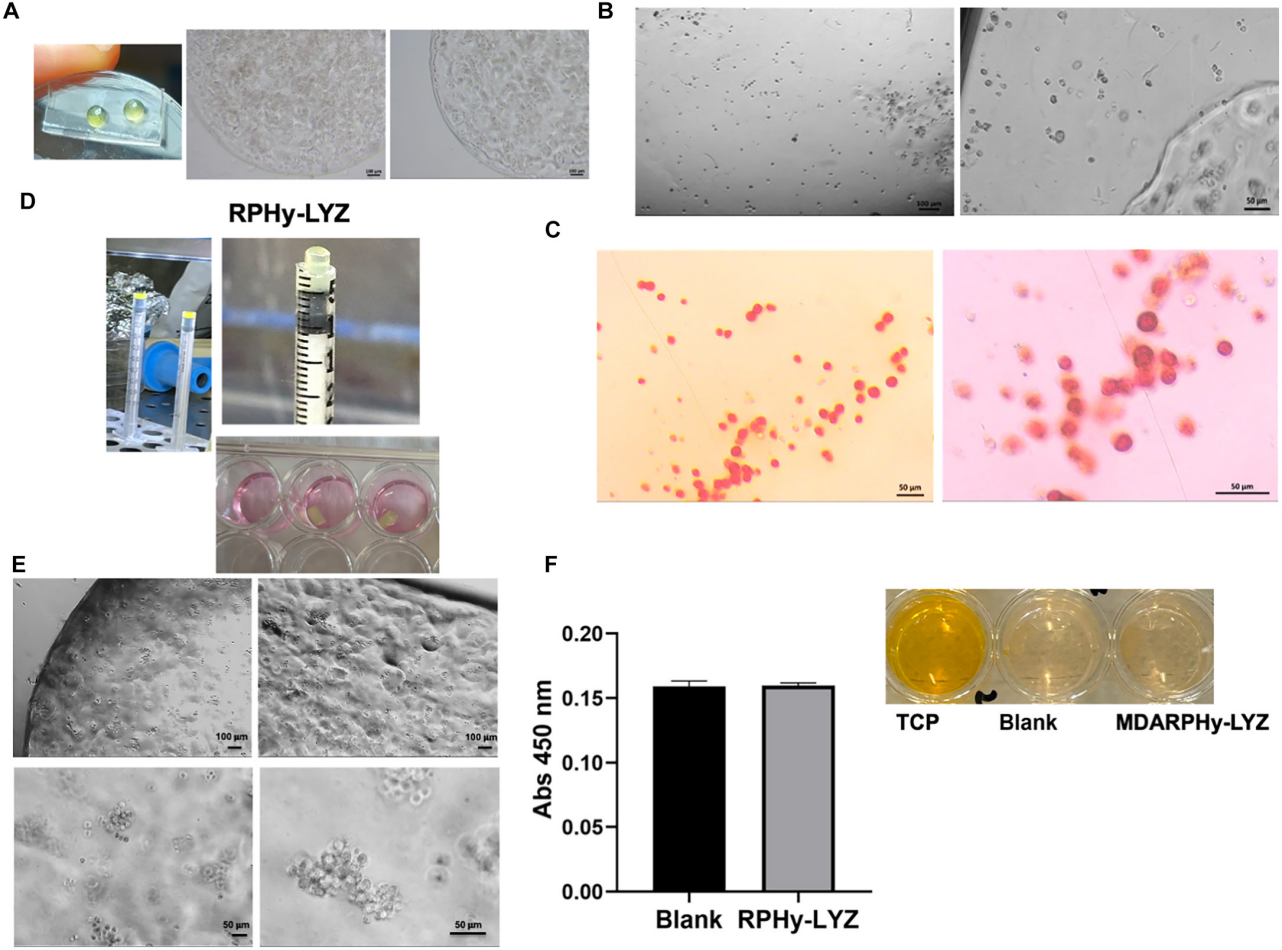
RP hydrogels as system for MDA-MB 231 embedding. (A) Bright-field micrographs and digital images of RPHy microspheres embedding MDA-MB 231 breast cancer cells (cell density of 10^4^ cells/μl) produced on a nanostructured superhydrophobic surface. (B) Bright-field micrographs of cancer cells extracted from the RPHy spheres and replated on a tissue culture plate (TCP). (C) Alizarin Red S staining of the calcium-rich deposits produced by the cancer cells following their growth in the hydrogel sphere and their subsequent extraction and replating. (D) Digital images of RPHy-LYZ polymerized under visible light using syringes as molds and of 3D MDARPHy-LYZ constructs in the cell culture medium. (E) Bright-field micrographs of MDARPHy-LYZ (cell density of 1.75 × 10^3^ cells/μl); (F) WST-1 cell viability assay of RPHy-LYZ without cells (blank) and with cells (RPHy-LYZ) after 24 h of cell growth. Experiments were performed as 3 independent replicates. The error bar indicates SD. Scale bars are 100 and 50 μm.

MDARPHy microspheres were produced using a nanostructured superhydrophobic surface [18] (see Fig. S3) and embedding MDA-MB 231 cancer cells during the LED photopolymerization of the precursor mixture of RPHy (Fig. 9A). MDARPHy spheres were cultured for 7 d in the cell culture medium (Fig. 9A). The cells showed a globular morphology; therefore, the cells changes were assessed by extracting the cells from MDARPHy microspheres by using an optimized protocol and the extracted cells were reseeded. The cells showed good adhesion to the plate and were cultured for 6 d (Fig. 9B). However, a change in cell morphology was observed compared to MDA-MB 231 cells grown only on a plate. Considering the globular cell morphology and the high stiffness of the system of growth, it was investigated whether the tumor cells had undergone a transition to osteoblast-like cells. Alizarin Red staining was performed (see Fig. 9C), observing the production of calcium-rich deposits by cells derived from MDARPHy microspheres. Although more detailed cellular characterization is necessary, these data can indicate a transdifferentiation of MDA-MB 231 cells into osteoblast-like cells.

The transformation of breast cancer cells into osteoblasts has recently been observed and is referred to as epithelial–mesenchymal transition [18,29]. Increased calcium concentrations, calcium oxalate, and hydroxyapatite accumulations in tumors with metastasis were detected in tissue biopsy samples. However, only in recent years have the molecular mechanisms of these cellular transitions come to light. Therefore, the possibility of having a system able to induce the transition into osteoblast-like cells of breast cancer cells can open the way to studying in detail both the molecular mechanism of this physiological transformation and the effects of these transformed cells on both normal and other cancer cells. RPHy-LYZ was also tested for the embedding of the cancer cells as shown in Fig. 9D and E, but after 24 h of cell growth, no cell viability was observed (Fig. 9F). The presence of cellular aggregates was observable in RPHy-LYZ, which could be linked to the induction of endogenous–exogenous LYZ polymerization by LED exposure. These data highlight the possibility of using our LED-photopolymerizable RPHys for cell cancer embedding for therapeutical application.

## Discussion

**Fig. 10.**
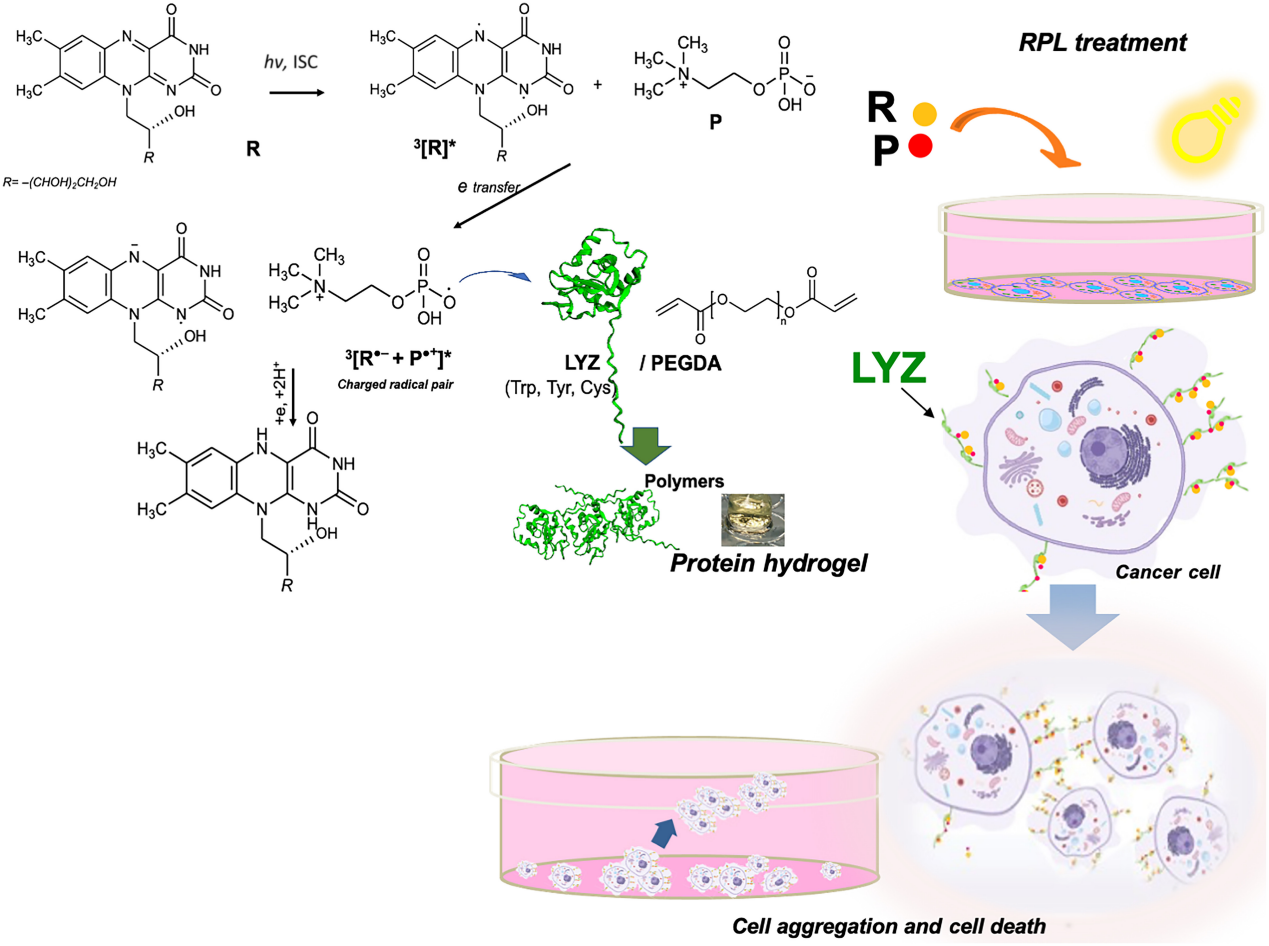
Scheme of the proposed photopolymerization reaction of the RPL system and of its antitumor activity on breast cancer cells. Riboflavin (R) is excited by photon absorption (*hv*) and, by intersystem crossing (ISC), reaches an excited triplet state (^3^[R]*). An electron transfer from phosphocholine (P) to ^3^[R]* could generate an [R^•−^] anion–[P^•+^] cation charged radical pair (^3^[R^•−^ + P^•+^]*). The highly reactive P^•+^ could interact with LYZ or PEGDA, initiating the polymerization process. At the cellular level, RPL treatment can induce the polymerization of ectopic LYZ, leading to the formation of cellular aggregates with detachment from the plate and consequent cell death.

The unique properties of the R molecule are directly linked to its chemical structure, which determines its biological activity and creates extensive opportunities for its use as a driving force in PD and in the fabrication of functional materials through polymerization processes. The first structural part that establishes R’s membership in the flavin family is a 3-ring structure—7,8-dimethyl-10-alkylisoalloxazine. This essential fragment is responsible for redox processes followed by catalytic activity, absorption, and photosensitivity [30]. The redox functionality of R is particularly advantageous in both biological action and the preparation of biomaterials. The isoalloxazine ring can easily undergo reduction processes, creating a balance between 3 redox forms: the oxidized form, semiquinone (acceptor of 1 electron), and fully reduced form (acceptor of 2 electrons). The redox form determines the chemical–physical properties of R [1]. The photosensitivity in aqueous media and the presence of a ribitol tail with 4 hydroxyl groups, easily modified for incorporating functional molecules such as polymerization initiation sites [31], make R and derivatives excellent photo-initiators in a wide range of applications and multifunctional molecules [32,33]. Here, we studied the molecular and cellular effects of the combined presence of R, P, and visible light. Our molecular results by electrophoresis, microscopy, and fluorescence spectroscopy on the RPL treatment of proteins demonstrate the ability of this system to induce photopolymerization of specific proteins, such as LYZ, which shows a natural capability to form fibrils [34–36]. Potential interaction sites of LYZ for both P and R have been proposed on the basis of the 3D molecular models obtained by the AlphaFold program, which are in agreement with the quenching of LYZ’s intrinsic fluorescence when P is added in the solution. The aggregation induced by RPL treatment demonstrates that the system can be used for the photopolymerization process of natural polymers, such as proteins. Consequently, we evaluated whether the RPL system was also able to induce cellular aggregation. The effects on different types of cell lines were assessed here, demonstrating a selective effect of RPL treatment. The high levels of both P and LYZ in MDA-MB 231 breast cancer cells can reasonably provide a causative explanation of RPL treatment effects on these cells. Previous studies have reported an altered choline metabolism in malignant transformation, resulting in a notable increase in P levels in tumor cells compared to those in nontumoral cells [8–11]. Our results show significantly higher levels of P in MDA-MB 231 compared to nontumoral MCF-10A cells (Fig. 2D) and high levels of LYZ expression with the presence of high-molecular-weight forms of LYZ in breast cancer cells, not detected in stem cells (Fig. 6). These observations suggest that is the concomitant presence of high levels of P and LYZ, especially in its high-molecular-weight forms, that can justify the RPL treatment effects, since a cell-aggregation process driven by R, P, and LYZ can occur, leading to cancer cell death. In standard conditions of cell growth, the presence of R, P, or RP in the medium does not affect cell viability/proliferation, and no different behavior depending on cell type was observable. In contrast, the cell growth in the presence of visible light activates the electron transfer between P and R, thus inducing a selective cellular photo-aggregation mechanism. The effects on the cells were investigated here using several techniques. Confocal microscopy using ThS staining of MDA-MB 231 cells after RPL treatment demonstrated that the cells were able to form cellular aggregates with consequential detachment from the plate and cell death (see the scheme in Fig. 10). Both cardiac and bone-marrow-derived MSCs were not affected by RPL treatment, as shown by cell viability and microscopy analyses after treatment. The reason for the selective effect of RPL treatment on cancer cells, which was different from that on stem cells, can be found in the diverse composition of the extracellular matrix or cell surface. On the basis of our molecular results for LYZ, we evaluated the possibility that the effects of RPL treatment could be related to differences in LYZ expression between cancer cells and MSCs. The western blotting analyses (Fig. 6A) showed the presence of high levels of LYZ in the cancer cells; in particular, an LYZ form at a high molecular weight (about 50 kDa) was observed, corresponding to the trimeric form of the protein. Moreover, the confocal microscopy analyses showed the presence of LYZ in the cell aggregates obtained after RPL treatment. In contrast, the LYZ expression in BMMSCs was very low and no LYZ at a high molecular weight was observable. Therefore, in agreement with the LYZ polymerization in vitro induced by RPL, the cell–cell aggregates could be due to an LYZ cross-linking between the cells. These results are also in accord with recent studies that show a correlation between LYZ and human tumors. In a study involving 177 breast cancer tissue sections, it was found that 126 of these sections exhibited positive LYZ staining in tumor cells as detected by immunohistochemistry. Notably, a high LYZ staining score correlated with improved overall survival and relapse-free survival was observed, indicating that LYZ is a favorable prognostic factor in breast cancer patients [37]. Myeloid and leukemic cells and solid tumor cells also express LYZ, which correlates with tumor prognosis. Elevated levels of LYZ in serum and urine were previously observed in cases of monocytic and monomyelocytic leukemia [38], suggesting that LYZ could potentially serve as a biomarker for these leukemias and related kidney injury. More recently, bioinformatic studies investigating the immune signatures of melanoma, laryngeal squamous cell carcinoma, glioblastoma, and soft tissue sarcomas have indicated that the messenger RNA level of LYZ can act as an immune-related marker for predicting prognosis or response to immune therapies in these tumors [12,13]. Moreover, hepatocellular carcinoma (HCC) cells were also found to express and secrete LYZ, which appears to be regulated by cytokine-mediated signaling and Wingless-related integration site (WNT) signaling activation [39–41]. Recently, ectopic expression of LYZ in HCC tissues was suggested by Gu et al. [14]. The immunofluorescence results indicated that LYZ was also present on the surface of PLC/PRF/5 cells, which themselves could express and secrete LYZ. Therefore, it was suggested that the secreted LYZ binds to the outer surface of the tumor cells. Moreover, in the same study, the LYZ expression pattern in an external cohort’s HCC tissue microarray was determined through immunohistochemistry staining, and it was observed that high LYZ staining scores correlated with shorter overall survival in HCC patients. Therefore, the tumor progression in HCC cells can be related and promoted by the ectopic expression of LYZ [14]. Despite various studies examining the relationship between LYZ and tumors, the prognostic value and role of LYZ in tumors require further extensive investigations. The correlation, here observed, between RPL treatment sensitivity and ectopic LYZ expression in cells is further supported by the sensitivity of BMMSCs to RPL treatment following pretreatment with LYZ (Fig. 6C). The efficacy of RPL treatment was also tested here on 3D cultures of MDA-MB 231 cells (Fig. 7), demonstrating effects similar to those observed in 2D cultures. The selective effect of RPL treatment on triple-negative cancer cells in 2D and 3D cultures may open the way to a new tumor therapeutical approach. In contrast, the simple treatment of cells with R in light conditions of cell growth induces significant effects on the cell viability of all used cell lines. The light activation of R can lead to the production of free radicals, which induce oxidative damage in cells and programmed cell death.

However, the effects were higher in cancer cells than in stem cells. These differences may be related to the high endogenous P concentration in MDA-MB 231 cancer cells, which was demonstrated here by NMR spectroscopy studies. The oxidative damage in cancer cells can be combined with the protein and/or cellular aggregation induced by RPL treatment using endogenous P. This hypothesis can be supported by clinical studies, using ^1^H magnetic resonance spectroscopy, which have revealed that breast carcinoma lesions exhibit an intense signal absent in most benign breast lesions and normal breast tissue [42]. This resonance signal has a typical frequency of the trimethylamine group of choline and its metabolites, thus reflecting the metabolic changes in choline during neoplastic transformations of breast tissue [42]. Elevated levels of P and glycerophosphocholine have been observed in human breast carcinoma cells compared to normal HMECs [43]. Furthermore, by ^31^P magnetic resonance spectroscopy, high levels of phosphomonoesters have been detected in human breast neoplasia biopsies and in vivo. Increased choline uptake and rapid phosphorylation have been also identified in human breast carcinoma T47D cells and MCF7 cells [44]. Therefore, our results pave the way for studying new aspects of both the molecular mechanism underlying PDT using R and the physiological role of P. The potential photopolymerizing action of the RPL system on PEGDA was also investigated here for embedding of cancer cells. The protocols for obtaining 2 new visible-light-photopolymerizable PEG hydrogels (RPHy and RPHy-LYZ) were developed and optimized here. The photo-initiating functionality of R and derivatives has been extensively studied in the radical polymerization of acrylamides [45] as valid visible light-sensitive photo-initiators in the presence of electron donors (tertiary amines) as mild reducing agents [46]. Usually, R is a type II initiator, where the photoexcited initiator reacts with the co-initiator, i.e., electron acceptor or donor, or hydrogen donor, generating radicals or radical ions. The photoexcited triplet state of the R molecule accepts an electron from the amine, generating the long-lived neutral amine radical, thereby initiating the polymerization of free radicals [45]. Further development of R-mediated radical polymerization has been through the synthesis of poly(meth)acrylates [31,47]. For the first time, P was used here as an electron donor in the photopolymerization process of PEGDA induced by R (see the scheme in Fig. 10), making this process more sustainable for 3D cell culture system production [18,19,21] and bioprinting tissue technology and showing potential relevant physiological implications. RPHy and RPHy-LYZ exhibited equivalent antimicrobial activity against ampicillin-resistant bacteria (Fig. 8C), which was also indicative of LYZ polymerization and, consequently, loss of enzymatic activity. The hydrogels were characterized here for their capability to embed cancer cells, observing a transdifferentiation into osteoblast-like cells of MDA-MB 231 cells embedded in RPHy, probably induced by the hydrogel stiffness for a physically induced transdifferentiation [18], and cell death using RPHy-LYZ. This result is also in agreement with the increase in cell death induced by the presence of LYZ in association with RPL treatment. LED photopolymerization on the skin of the hydrogels was also demonstrated here, suggesting that a potential selective treatment of tumors using the RPL system in either solution or hydrogel form may be exploited.

Moreover, since new PDT and PTT antitumor strategies are emerging [48–50] and new hydrogel treatments exploiting PDT/PTT are gaining increasing interest, the here produced RPHy hydrogels could represent suitable tools for therapeutic applications. Despite RPL treatment and RPHy holding great potential for clinical use, still some limits need to be addressed to scale up the systems in cancer therapy. Firstly, in vivo experiments are required in order to more completely assess the efficacy of RPL treatment. Furthermore, to extend this treatment to other cancer types, the variability in P levels and in LYZ expression among different cell types should be investigated with specific focus on ectopic LYZ expression on the cell surface. Lastly, the observed physically induced transdifferentiation of breast cancer cells into osteoblast-like cells after cell embedding into RPHy needs further investigation. Several studies have demonstrated the presence of microcalcifications in breast tumors associated with poor prognosis and have found that the osteoblast-like features of breast cancer cells are correlated with increased metastasis to bone and increased tumor resistance to the multidrug therapies commonly used in breast cancer treatment [29]. However, in our previous studies [18], we transdifferentiated MDA-MB 231 breast cancer cells into osteoblast-like cells by embedding them into a stiff hydrogel, and a decreased expression of multidrug resistance glycoprotein P (Pgp) with an increased sensitization to doxorubicin was found for osteoblast-like cells compared to untransdifferentiated cells [18]. This suggests that, although the osteoblast-like cells exhibit reduced drug resistance, the production of biochemical factors (e.g., Ca^2+^, matrix metalloproteinases, and microRNAs) may stimulate untransdifferentiated cancer cells to enhance their proliferation and matrix metalloproteinase production, leading to an increase in cell migration and metastasis. Therefore, the observed transdifferentiation of breast cancer cells into osteoblast-like cells after cell embedding into RPHy may represent a viable strategy in cancer treatment when in situ gel polymerization is followed by localized treatment with an anticancer drug. Consequently, our results pave the way for exploiting the physiological increase in P and LYZ in cancer cells as a potential Achilles’ heel of tumor cells.

## Data Availability

The authors declare that all data generated in this study are available within the article or the Supplementary Materials. Other data related to this work are available from the corresponding author upon request.

## Supplementary Materials

Supplementary 1 Figs. S1 to S3 Video S1 AlphaFold original files
