# Supplementary material for: A New Light-Sensor System Affecting Cancer Cell Fate
Source: Biomater Res. 2025 Mar 5;29:0157. doi: 10.34133/bmr.0157 (PMC11880576; doi:10.34133/bmr.0157)
Supplement: Supplementary 1 — Figs. S1 to S3 Video S1 AlphaFold original files [file bmr.0157.f1.zip › original experimental data.pptx]

## Slide 1
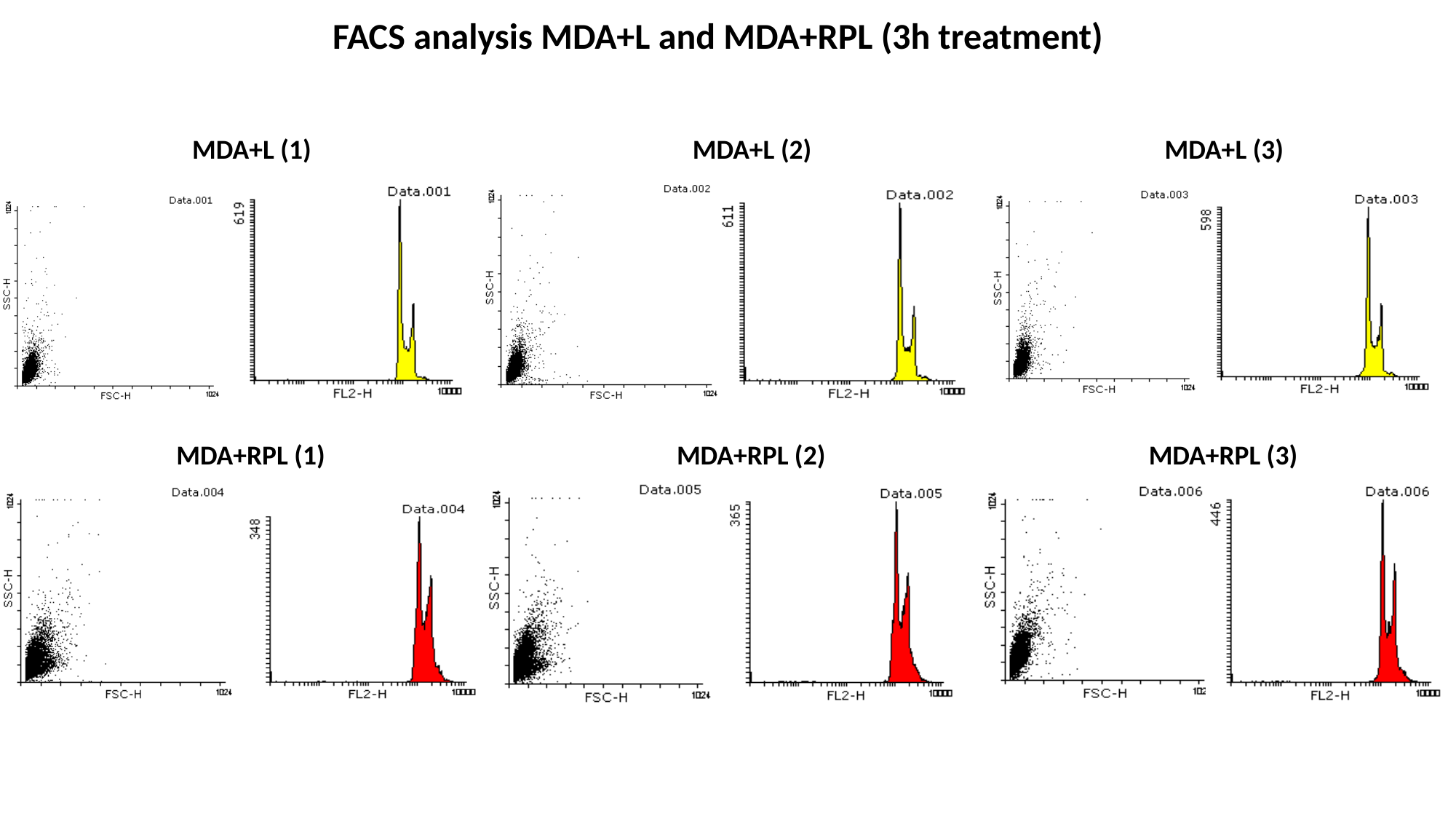

FACS analysis MDA+L and MDA+RPL (3h treatment)
MDA+L (1)
MDA+L (2)
MDA+L (3)
MDA+RPL (1)
MDA+RPL (2)
MDA+RPL (3)

## Slide 2
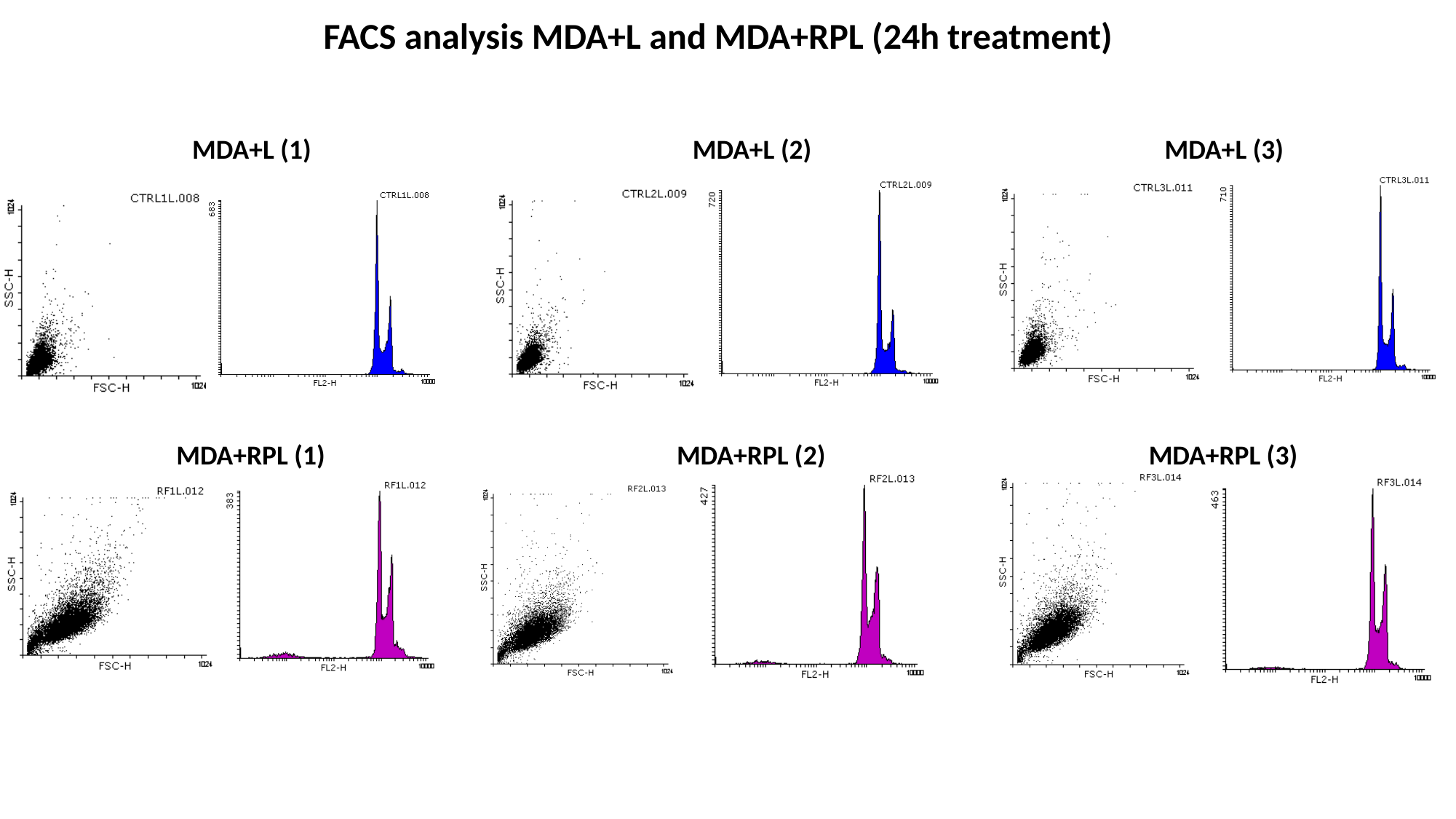

FACS analysis MDA+L and MDA+RPL (24h treatment)
MDA+L (1)
MDA+L (2)
MDA+L (3)
MDA+RPL (1)
MDA+RPL (2)
MDA+RPL (3)

## Slide 3
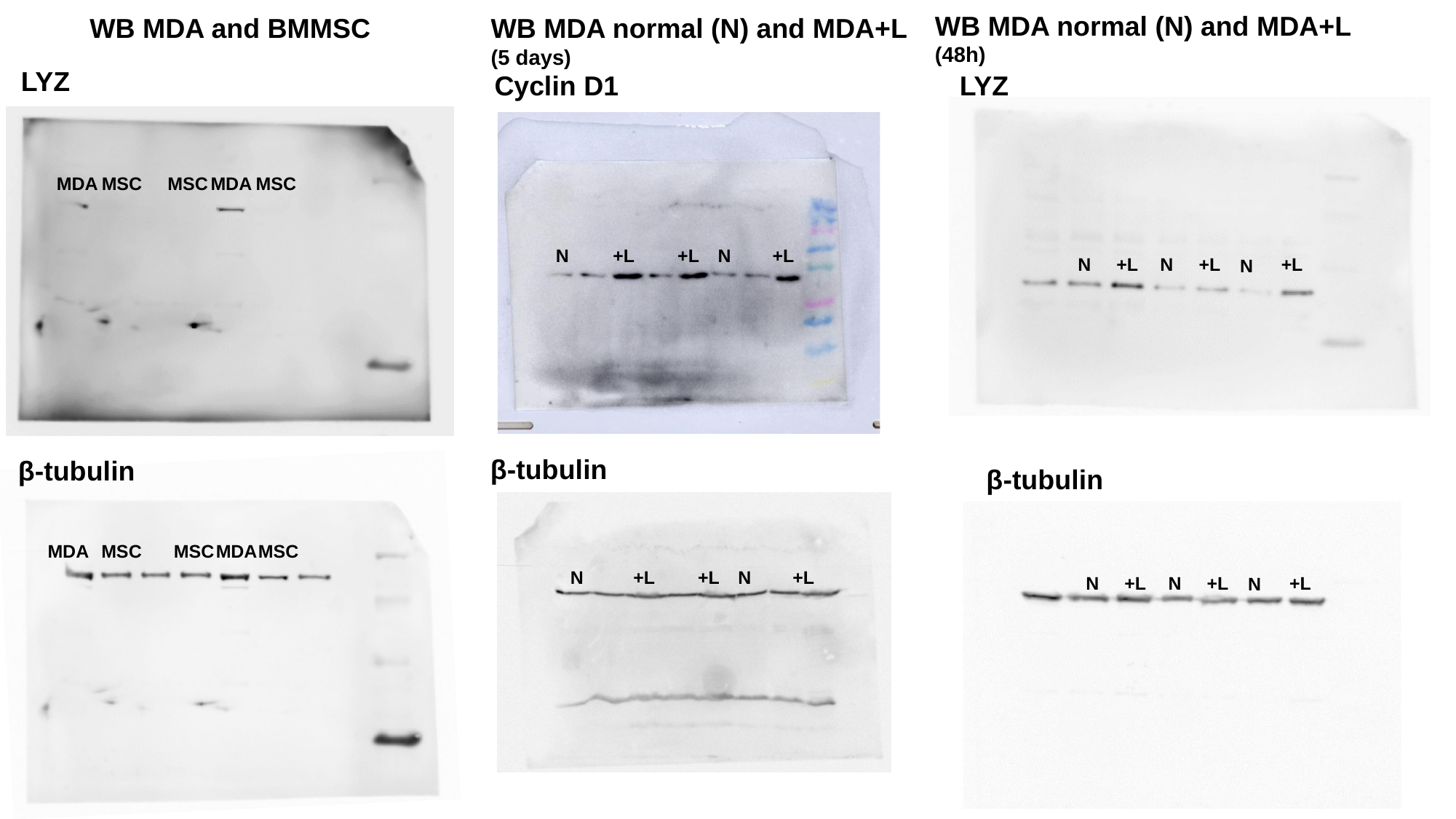

WB MDA normal (N) and MDA+L
(48h)
WB MDA and BMMSC
WB MDA normal (N) and MDA+L (5 days)
LYZ
MSC
MSC
MSC
MDA
MDA
β-tubulin
MSC
MDA
MDA
MSC
MSC
Cyclin D1
N
+L
+L
N
+L
β-tubulin
N
+L
+L
N
+L
LYZ
N
+L
N
+L
+L
N
β-tubulin
N
+L
N
+L
+L
N

## Slide 4
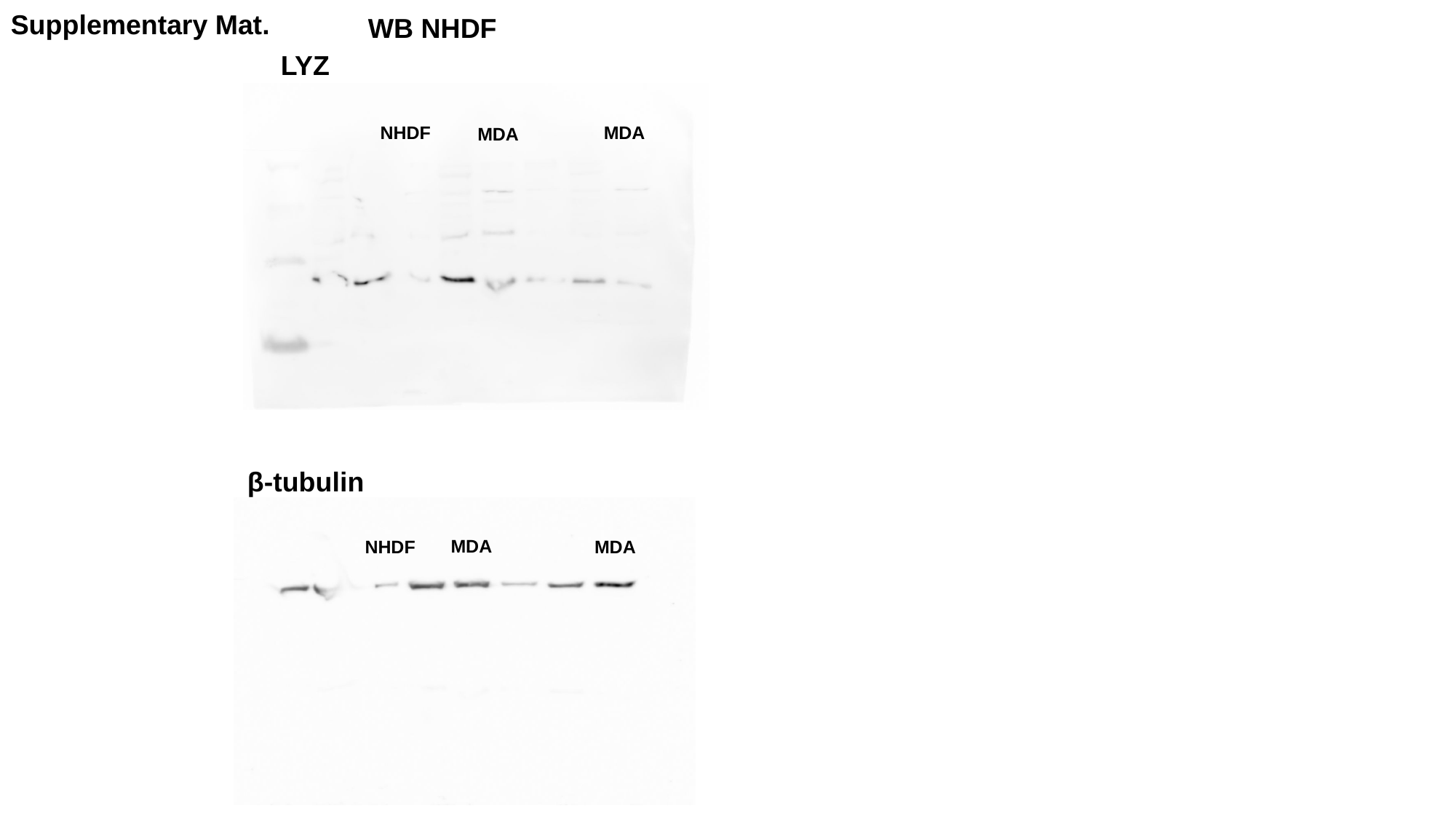

Supplementary Mat.
WB NHDF
LYZ
MDA
NHDF
MDA
β-tubulin
MDA
MDA
NHDF
