# Supplementary material for: A New Light-Sensor System Affecting Cancer Cell Fate
Source: Biomater Res. 2025 Mar 5;29:0157. doi: 10.34133/bmr.0157 (PMC11880576; doi:10.34133/bmr.0157)
Supplement: Supplementary 1 — Figs. S1 to S3 Video S1 AlphaFold original files [file bmr.0157.f1.zip › Supplemental Information Buonvino S et al 2024 .docx]

**Supplemental Information**

**A new Light-Sensor system affecting cancer cells fate.**

Silvia Buonvino, Ilaria Arciero, Stefano Moretti, Egidio Iorio and Sonia Melino^*^

**Supplementary Figures**

**
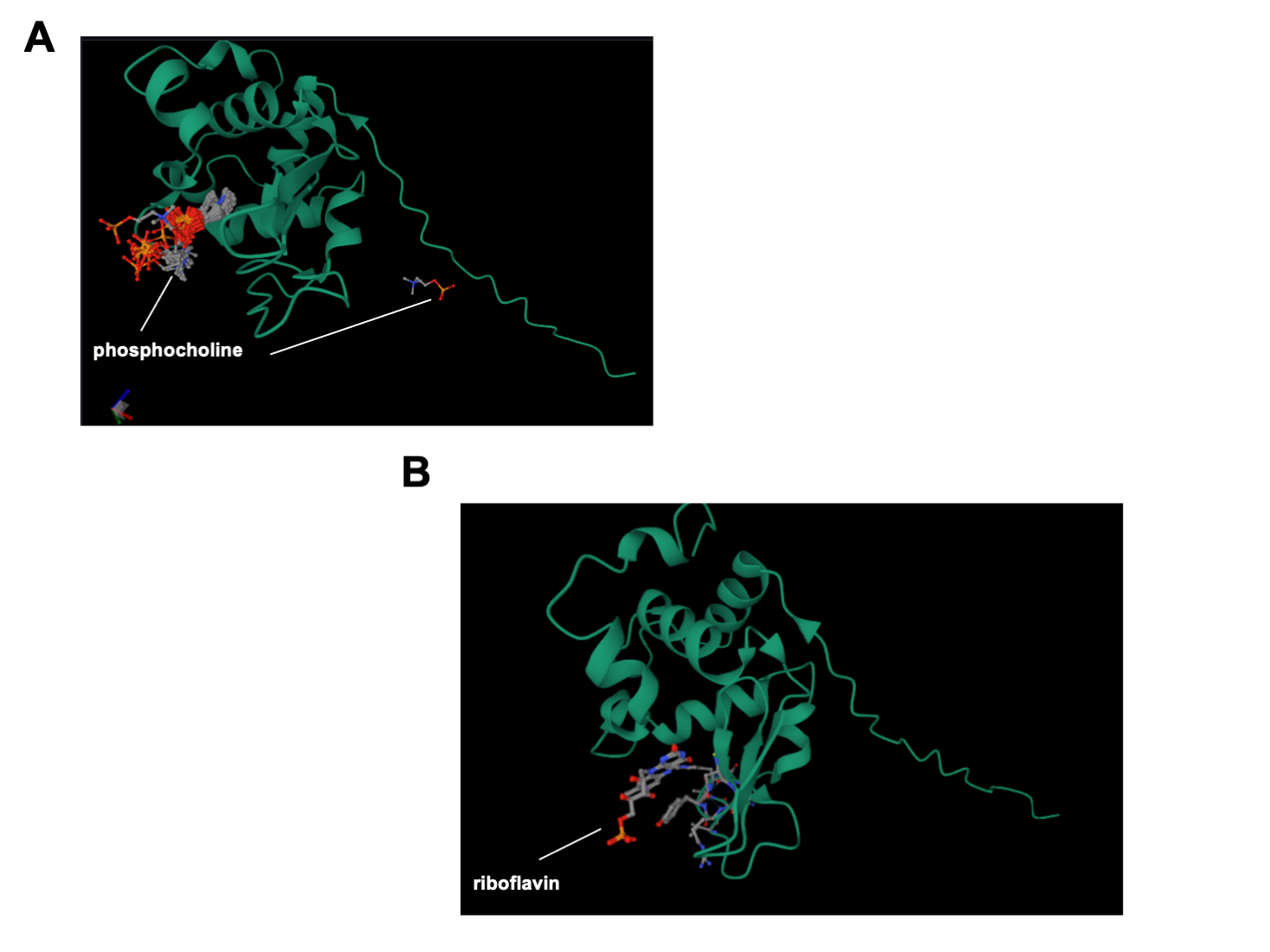
**

**Figure 1S.** 3D LYZ structure with the predicted interaction sites of phosphocholine (P) and riboflavin (R) obtained using AlphaFold 2 program and DiffDock-L.

**
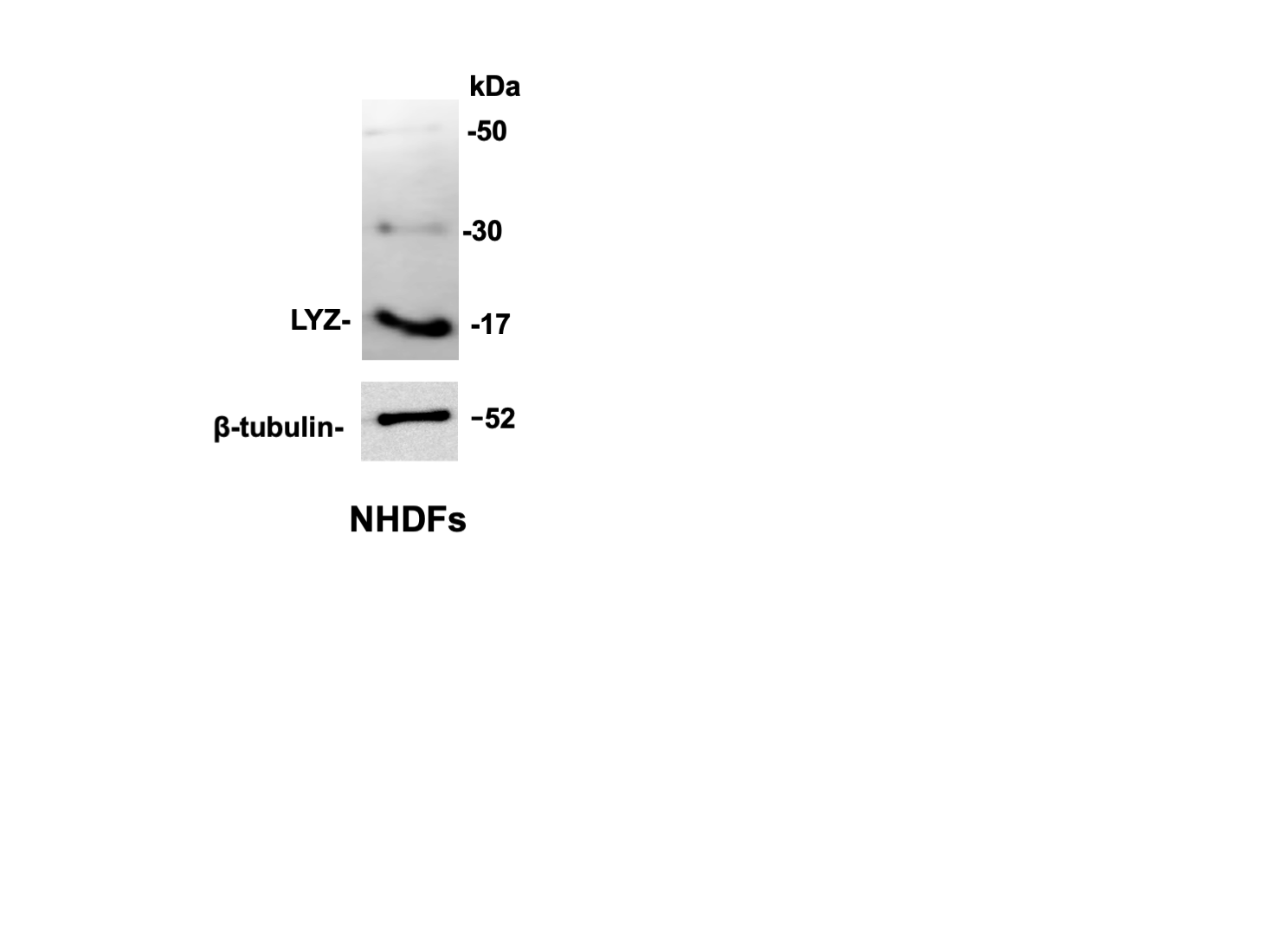
**

**Figure 2S.** LYZ expression in NHDFs by western- blot analysis.


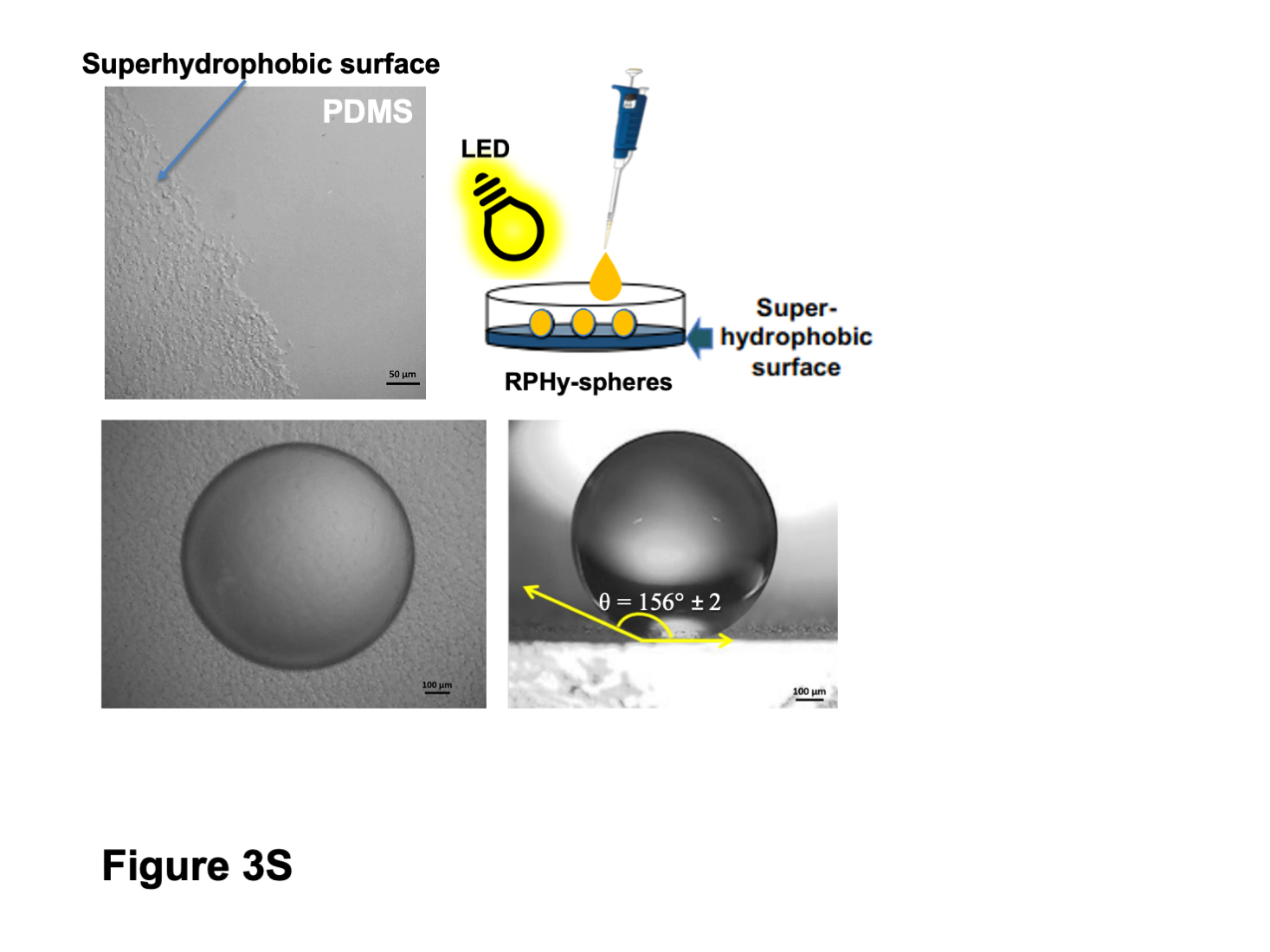


**Figure 3S.** Micrographs of the superhydrophobic surface and schematic representation of the production of the microspheres of MDARPHys.
