# Supplementary figures and images for: A New Light-Sensor System Affecting Cancer Cell Fate
Source: Biomater Res. 2025 Mar 5;29:0157. doi: 10.34133/bmr.0157 (PMC11880576; doi:10.34133/bmr.0157)

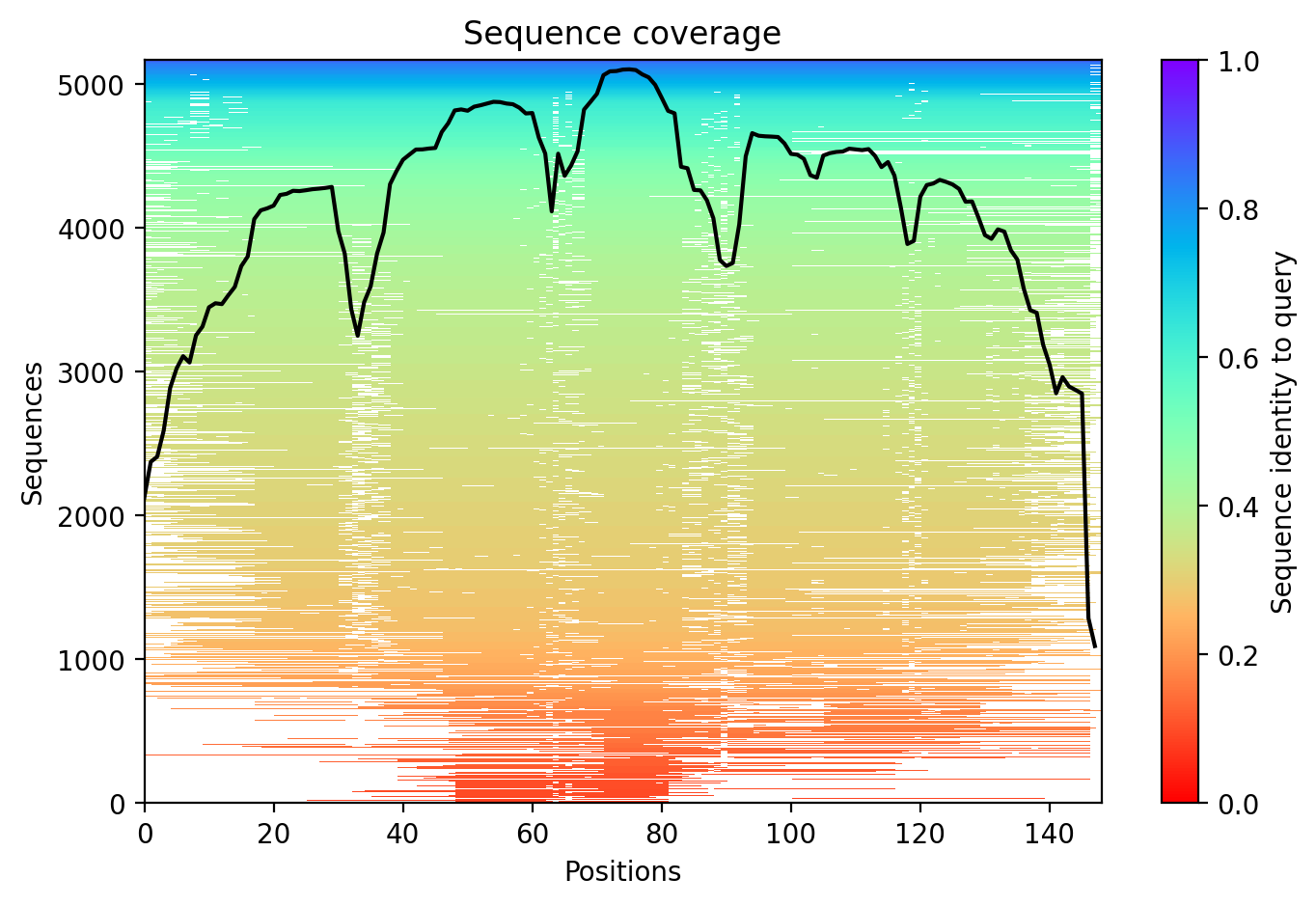

Supplement: Supplementary 1 — Figs. S1 to S3 Video S1 AlphaFold original files [file bmr.0157.f1.zip › 1_coverage.png]
